# Modification and Synergistic Studies of a Novel Frog Antimicrobial Peptide against *Pseudomonas aeruginosa* Biofilms

**DOI:** 10.3390/antibiotics13070574

**Published:** 2024-06-21

**Authors:** Xinze Liu, Daning Shi, Shiya Cheng, Xiaoling Chen, Chengbang Ma, Yangyang Jiang, Tao Wang, Tianbao Chen, Chris Shaw, Lei Wang, Mei Zhou

**Affiliations:** 1 Natural Drug Discovery Group, School of Pharmacy, Queen’s University Belfast, Belfast BT9 7BL, UK; xliu54@qub.ac.uk (X.L.); scheng09@qub.ac.uk (S.C.); xiaoling.chen@qub.ac.uk (X.C.); c.ma@qub.ac.uk (C.M.); t.chen@qub.ac.uk (T.C.); c.shaw@qub.ac.uk (C.S.); l.wang@qub.ac.uk (L.W.); m.zhou@qub.ac.uk (M.Z.); 2 Chinese Academy of Agricultural Sciences, No. 12 Zhongguancun South Street, Haidian District, Beijing 100081, China; shidaning@caas.cn

**Keywords:** antimicrobial peptides (AMPs), peptide modification, antibiotics, co-administration, *Pseudomonas aeruginosa*

## Abstract

The overuse of traditional antibiotics has resulted in bacterial resistance and seriously compromised the therapeutic efficacy of traditional antibiotics, making the exploration of new antimicrobials particularly important. Several studies have shown that bioactive peptides have become an important source of new antimicrobial drugs due to their broad-spectrum antibacterial action and lack of susceptibility to resistance. In this study, a novel bioactive peptide Nigrosin-6VL was characterised from the skin secretion of the golden cross band frog, *Odorrana andersonii*, by using the ‘shotgun’ cloning strategy. Modifications on the Rana Box of Nigrosin-6VL revealed its critical role in antimicrobial functions. The peptide analogue, 2170-2R, designed to preserve the Rana Box structure while enhancing cationicity, exhibited improved therapeutic efficacy, particularly against Gram-negative bacteria, with a therapeutic value of 45.27. Synergistic studies demonstrated that 2170-2R inherits the synergistic antimicrobial activities of the parent peptides and effectively enhances the antimicrobial capacity of cefepime and gentamicin against both planktonic cells and biofilms. Specifically, 2170-2R can synergise effectively with cefepime and gentamicin against different strains of *P. aeruginosa* biofilms. Consequently, 2170-2R holds promise as a potent antimicrobial agent developed to combat infections induced by *Pseudomonas aeruginosa*.

## 1. Introduction

For over half a century, the wide applications of antibiotics have greatly improved our ability to treat bacterial infections all around the world [1]. However, antibiotic resistance poses a significant hazard to human health due to incorrect antibiotic usage in clinical settings, which urgently requires us to develop novel treatment options and alternative antimicrobial therapies [2,3,4]. Meanwhile, traditional antibiotics may also cause adverse host reactions [5]. Therefore, discovering new antimicrobials with low toxicity or boosting the antibacterial activity of current antibiotics through synergistic applications is considered an efficient strategy, which also has the potential to improve drug safety and efficacy. Many studies have demonstrated that bioactive peptides exhibit antimicrobial activity while being less susceptible to drug resistance because of their unique membrane destruction mechanism [6,7]. In addition, some of them show antioxidant, anticancer, anti-inflammatory, and other properties, making them an excellent source of novel antimicrobials [8,9,10]. Consequently, research on AMPs has gained great attention in the past decades, and the development of AMPs has been considered an effective direction to address the resistance problems [11].

As a widespread Gram-negative opportunistic bacterium, Pseudomonas aeruginosa strains have become one of the most common causes of nosocomial infections due to their many phenotypes and metabolism [12]. *P. aeruginosa* is increasingly resistant to conventional antibiotics, which can be compounded by the formation of biofilms on surfaces, conferring additional resistance [13]. Once *P. aeruginosa* produces biofilms to create chronic infections, it undergoes a sequence of changes that aggravate multidrug resistance, making difficult-to-treat infections potentially fatal, particularly in people with the hereditary condition cystic fibrosis and other immune weaknesses [14,15,16].

Antibiotic resistance management aims to slow the rate of resistance, and it requires the use of more effective infection remedies in addition to restricting the usage of antibiotics [17]. Such restrictions are mostly encountered in combinatory medicines, which, unlike monotherapies, use many pharmaceuticals to treat a single disease at the same time. Combining different medications results in either synergism or antagonism. A synergistic reaction occurs when a combination of medications has a significantly stronger effect than single treatments, going beyond an additive effect [17]. Peptides used in combination with antibiotics may have an inhibitory effect on antibiotic-resistant bacteria. For example, studies have shown that the antibiotic-resistant bacteria *P. aeruginosa* can be eradicated by combining the peptide DP7 with azithromycin or vancomycin [18]. Similarly, the peptide SET-M33 was found to be extremely effective against a wide range of multidrug-resistant organisms, such as *P. aeruginosa*, especially when paired with rifampin [19]. In addition to being effective against planktonic bacteria, the combination of peptides and antibiotics is also favourable for improving the inhibition of bacterial biofilms. Based on the studies from Rudilla et al., a recent evaluation of AMP38, a new synthetic cyclo-lipopeptide analogue of polymyxin, in combination with carbapenems, revealed a synergistic effect able to kill *P. aeruginosa*, which forms biofilms and is resistant to carbapenems [20].

Several Nigrosin peptides have been discovered in various amphibian species. These peptides primarily act on Gram-positive and -negative bacteria, while some also have antiviral and antifungal effects. The most notable structural feature of the Nigrosins is the Rana Box, characterised by a seven-element loop structure linked by a disulphide bridge at the C-terminus [21,22,23,24]. However, the role of the Rana Box is still ambiguous and remains controversial. Some believe that this structure is not necessarily the key structure that determines the effect of the AMPs [25,26]. In this study, a new Nigrosin peptide, Nigrosin-6VL, was identified from the skin secretion of the golden cross band frog, *Odorrana andersonii*. Given the controversial role of the Rana Box, we first explored the effects of this loop structure on the antimicrobial activities of Nigrosin-6VL. Subsequently, we employed cationic enhancement and successfully developed an analogue, peptide 2170-2R, with improved therapeutic potential. Not only did peptide 2170-2R demonstrate stronger antimicrobial activities compared to the natural template but it also inherited the low toxicity and synergistic antimicrobial properties of the parent peptide. Peptide 2170-2R greatly improved the antimicrobial efficacy of cefepime and gentamicin, particularly against *P. aeruginosa* strains.

## 2. Results

### 2.1. ‘Shotgun’ Cloning of Peptide Nigrosin-6VL Biosynthetic Precursor Complementary DNA (cDNA)

**Figure 1:**
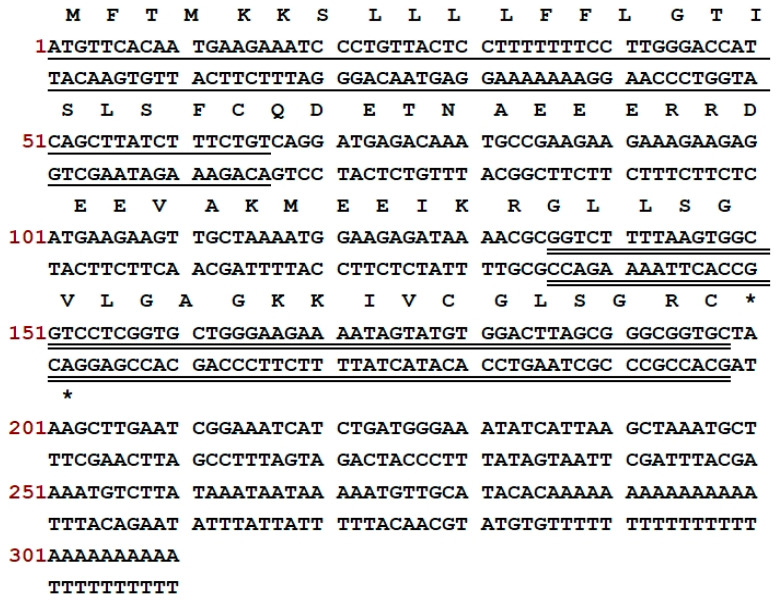
The open reading frame nucleotide and translated sequences, as well as the deduced sequence of Nigrosin-6VL. The nucleotide sequence of the probable signal peptide is single-underlined, the nucleotide of the mature peptide is double-underlined, and the asterisk (*) indicates a stop codon. The numbers in red indicate the positions of nucleotides.

**Figure 2:**
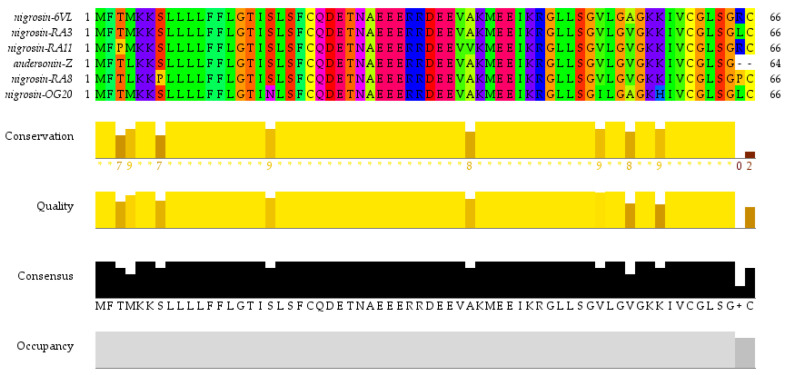
Descriptions and comparison of the peptide precursors of five selected analogues from the Nigrosin family. Multiple alignments of Nigrosin-6VL peptide precursors and homologues. An asterisk (*) indicates that the location has a highly conserved residue. Taylor colour scheme is used to label and reveal patterns of variations.

*Odorrana* species are renowned for their rich diversity and abundance of AMPs [27]. As a representative *Odorrana* species, *O. andersonii* was, therefore, the focus of this work. Through using the ‘shotgun’ cloning strategy, a precursor cDNA encoding an AMP was identified from the skin secretion of *O. andersonii*. As illustrated in Figure 1, the precursor contained 66 amino acids separated into three segments: a potential signal sequence of 22 amino acids, a mature sequence of 21 amino acids, and an acidic spacer region. The amino acid symbol “-KR-” (-Lys-Arg-) suggested a typical cleavage location. The putative sequence of the mature peptide is GLLSGVLGAGKKIVCGLSGRC. Sequence alignment employed in the NCBI-BLAST programme demonstrated that this is a novel peptide and it shares high similarity with peptides from the Nigrosin family (Figure 2). The new peptide was named Nigrosin-6VL. The nucleotide sequence of the Nigrosin-6VL precursor was submitted to GenBank with an accession number of PP757549 (accession on 11 May 2024). Although Nigrosin-6VL shares a high degree of similarity with other Nigrosins, the previous literature has indicated that structural differences in AMPs from *Odorrana* frogs can lead to remarkably variations in antimicrobial activity [27]. Therefore, Nigrosin-6VL was chosen for further research.

### 2.2. Predicted Secondary Structure and Physicochemical Characteristics of the Original Peptide Nigrosin-6VL

**Figure 3:**
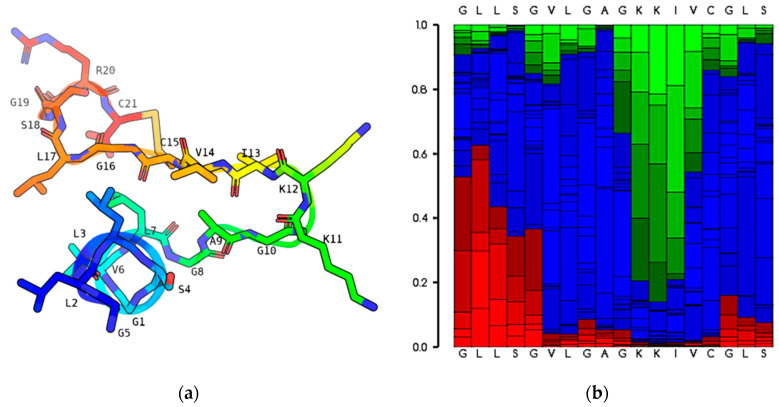
The predicted configuration of Nigrosin-6VL when analysed by the PEPFOLD3 server. (**a**) The 3D model of the predicted Nigrosin-6VL. (**b**) The possible distribution of the secondary structure of each residue. Red represents helical, blue represents coil, and green represents extended.

As shown in Figure 3, the predicted secondary structure from the PEPFOLD3 programme indicated that the peptide Nigrosin-6VL showed a predominately extended structure with a loop (Rana Box) formed by cysteine (C) in the 15th and 21st positions at the peptide’s C-terminus. The molecular mass of the peptide was 1986.088 Da and it carried three positive charges contributed by two lysine (K) residues and one arginine (R) residue. The grand average hydropathy value of the peptide (GRAVY) was 0.886, revealing that the peptide Nigrosin-6VL was more hydrophobic.

### 2.3. Physicochemical Properties and Structural Modification of Nigrosin-6VL

As previously indicated, the peptide Nigrosin-6VL’s secondary structure was separated into the Rana Box at the C-terminus and the predominant extended structure at the N-terminus. Therefore, the seven modified peptides were obtained from two directions. The modified peptides 1885-C, 1885-R, 1924-A, and 1782-R were obtained by Rana Box modifying, specifically removing one or two Cys residues in the 15th and 21st positions and replacing them with Ala. The other three peptides were created by structurally modifying the N-terminus, mostly by replacing the amino acid Gly with Ala to promote potential helicity or by adding the amino acid R (Arg) to enhance the number of positive charges. The sequences and specific characteristics of Nigrosin-6VL and the designed analogues are shown in Table 1. All peptides were synthesised using the SPPS strategy. The synthetic peptides were characterised and purified to achieve a purity of over 90% for functional tests (Figure S1).

### 2.4. Anti-Planktonic Microorganism Activity Selection of Nigrosin-6VL and Its Analogues

The antimicrobial properties of Nigrosin-6VL and its analogues are presented in Table 2. The minimal inhibitory concentration (MIC) and minimal bactericidal concentration (MBC) of all peptides were measured against Gram-positive bacteria, Gram-negative bacteria, and yeast. The antimicrobial activity of the novel peptide Nigrosin-6VL was relatively weaker compared to many reported frog-derived AMPs. Compared to the parent peptide, the antimicrobial effects of modified peptides were improved remarkably. In general, these peptides were more effective in inhibiting Gram-negative bacteria, especially *E. coli* ATCC 8739. Among Gram-positive bacteria, *S. aureus* ATCC 6538 and MRSA NCTC 12493 were more easily killed by peptides. All peptides showed no significant inhibitory effect on yeast, *C. albicans* ATCC 10231.

### 2.5. Haemolysis Activity and TI Values of Nigrosin-6VL and Its Analogues

As shown in Table 3, the peptides had no significant haemolytic effect in the experimental concentration range, and the highest haemolysis was also less than 10%. For most tested peptides, the TI values of tested Gram-negative bacteria were higher than the tested Gram-positive ones. Among the tested peptides, the analogue 2170-2R showed the highest TI values (45.27) against Gram-negative bacteria.

### 2.6. Time-Killing Kinetic Studies of Nigrosin-6VL and 2170-2R

**Figure 4:**
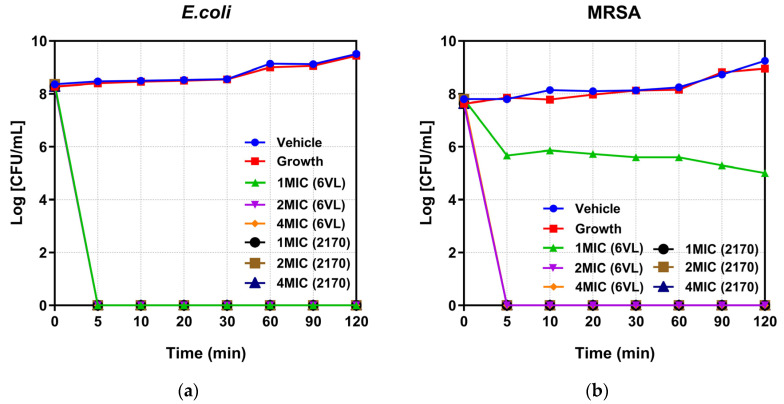
The kinetic killing curves of peptides Nigrosin-6VL and 2170-2R against *E. coli* ATCC 8739 (**a**) and MRSA NCTC 12493 (**b**) at 1, 2, and 4× MIC. Nigrosin-6VL is abbreviated as 6VL, and 2170-2R is abbreviated as 2170 in the figure. The error bar represents the standard error of the mean (SEM) calculated from nine replicates obtained from three separate experiments. Since Nigrosin-6VL did not exhibit antibacterial activity within the tested range, we assumed its highest tested concentration of 128 μM as its MIC for comparison purposes. Consequently, in the figure, the 1×, 2×, and 4× MIC values for Nigrosin-6VL correspond to actual concentrations of 128 μM, 256 μM, and 512 μM, respectively.

Two types of bacteria, *E. coli* ATCC 8739 and MRSA NCTC 12493, were selected for time-killing kinetic studies for the peptides Nigrosin-6VL and 2170-2R. According to the results in Figure 4a, both peptides killed *E. coli* ATCC 8739 at three concentrations, 1×, 2×, and 4× MIC, in 5 min. Figure 4b shows that peptide 2170-2R at the 1× MIC concentration can kill MRSA NCTC 12493 within 5 min. However, the original peptide, Nigrosin-6VL, only inhibited bacterial growth at the same concentration, merely eradicating bacteria at concentrations of 2× and 4× MIC.

### 2.7. Antimicrobial Mechanism Studies of Nigrosin-6VL and 2170-2R

#### 2.7.1. Effects on Bacterial Outer Membrane Permeability

**Figure 5:**
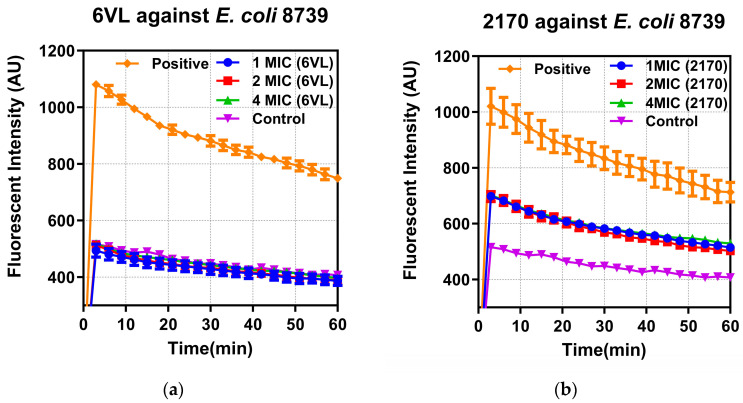
Fluorescent intensity of peptide Nigrosin-6VL (**a**) and 2170-2R (**b**) against *E. coli* ATCC 8739 at 1×, 2×, and 4× MIC in the NPN assay. Nigrosin-6VL is abbreviated as 6VL, and 2170-2R is abbreviated as 2170 in the figure. The control group was the bacteria treated with HEPES solution. Melittin at a concentration of 16 µM was used as the positive control. The error bar represents the standard error of the mean (SEM) calculated from nine replicates obtained from three separate experiments.

To explore the capacity of the peptides to disturb this lipid bilayer, the membrane permeability of *E. coli* ATCC 8739 was assessed using the fluorescent dye N-phenyl-1-naphthylamine (NPN). As illustrated in Figure 5, the peptide Nigrosin-6VL and the control group had similar fluorescent intensity values, indicating a weak permeabilisation ability of the outer cell membrane of bacteria. However, the values of the peptide 2170-2R and negative control were remarkably different. Furthermore, there was little change in peptide 2170-2R-induced permeability of the outer cell membrane as the concentration increased. In the dynamic experiment, the fluorescence intensity quenched naturally with time.

#### 2.7.2. Effects of Nigrosin-6VL and 2170-2R on Bacterial Intracellular Membrane Permeability

**Figure 6:**
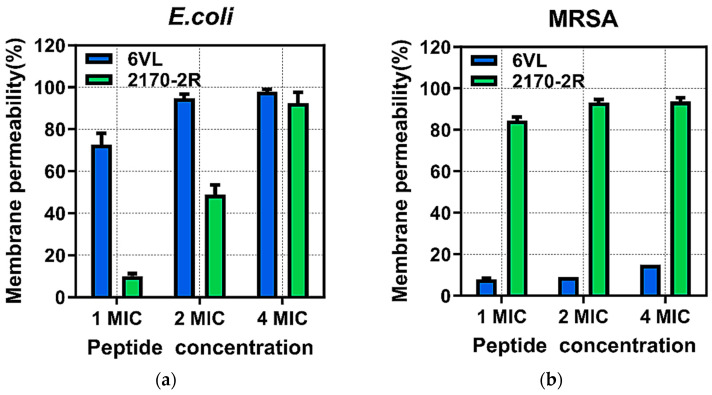
Permeability curves of peptides Nigrosin-6VL and 2170-2R against *E. coli* ATCC 8739 (**a**) and MRSA NCTC 12493 (**b**) at 1×, 2×, and 4× MIC. The total percentage (100%) of membrane permeabilisation was achieved by using melittin (20 μM). Nigrosin-6VL is abbreviated as 6VL, and 2170-2R is abbreviated as 2170 in the figure. The error bar represents the standard error of the mean (SEM) calculated from nine replicates obtained from three separate experiments.

To confirm the permeability mechanisms of the peptides Nigrosin-6VL and 2170-2R, *E. coli* ATCC 8739 and MRSA NCTC 12493 were, respectively, incubated with the peptides (1×, 2×, and 4× MIC) in the presence of SYTOX Green dye, and the fluorescence intensity was monitored. As shown in Figure 6, both peptides can induce a membrane permeability change in *E. coli* ATCC 8739 in a concentration-dependent manner. At concentrations of 4× MIC, both can induce around 100% permeability changes. For the tested MRSA strain, peptide 2170-2R induced approximately a 90% increase in permeability rate at the tested concentrations. In contrast, the changes in membrane permeability induced by the parent peptide Nigrosin-6VL remained at around 10% and did not increase with higher treatment concentrations.

### 2.8. Peptide and Antibiotic Co-Administration Studies

Previous research has demonstrated that peptides can permeabilise bacterial cell membranes, potentially contributing synergistically to the antimicrobial actions of antibiotics. In this study, four antibiotics with different antimicrobial mechanisms (cefepime, gentamicin, vancomycin, and levofloxacin) were selected for synergistic antibacterial experiments with peptides Nigrosin-6VL and 2170-2R. Firstly, the MIC values of four antibiotics against planktonic bacteria were determined (Table 4). A checkerboard assay was then used to detect the synergistic antimicrobial activity of peptides (Nigrosin-6VL and 2170-2R) and selected antibiotics, and the results of the co-administration are shown in Table 5. Among the tested antibiotics, cefepime and gentamicin showed relatively better synergistic effects with peptides. Therefore, they were the focus of our later studies.

### 2.9. Anti-Biofilm Studies

As shown in Table 6, the original peptide Nigrosin-6VL, and the most effective modified peptide 2170-2R, were selected to further explore their effects on biofilms. Compared to Nigrosin-6VL, 2170-2R exhibited relatively stronger effects on the tested biofilms.

### 2.10. Peptide and Antibiotic Co-Administration Studies of Anti-Biofilm Activities

Two antibiotics, cefepime and gentamicin, with the best antibacterial synergistic effects in MIC experiments were selected to carry out an MBIC assay with the combination of two peptides, Nigrosin-6VL and 2170-2R. In the first place, the MBIC and MBEC values for the peptides and antibiotics used alone were determined (Table 7). A checkerboard assay was subsequently performed to assess the synergistic anti-biofilm activity of peptides and antibiotics, and the findings of the co-administration assay are presented in Table 8.

**Figure 7:**
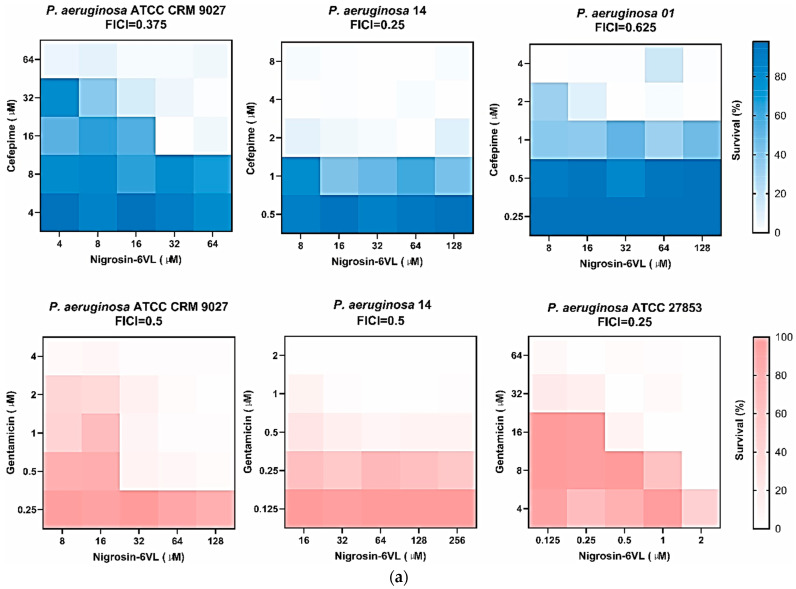

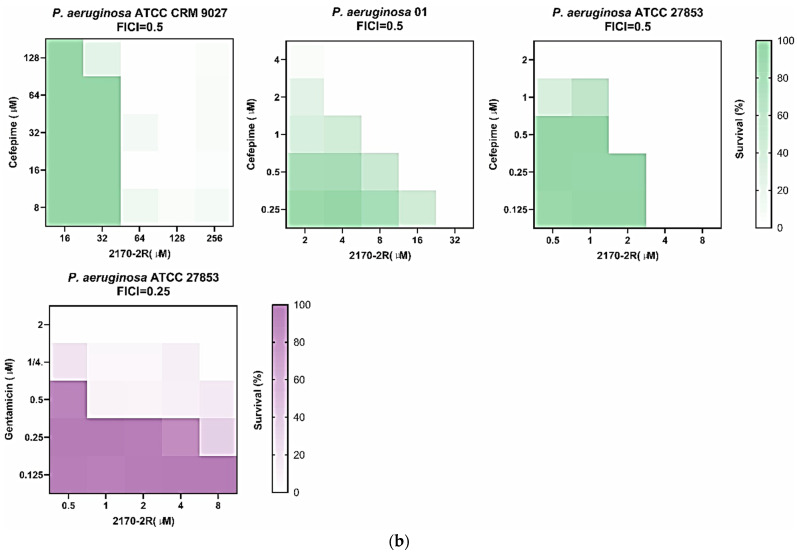
Heat plots of checkerboard assays for Nigrosin-6VL (**a**) and 2170-2R (**b**) in combination with cefepime and gentamicin against *P. aeruginosa* biofilm.

Since the synergistic inhibition of biofilms by peptides and antibiotics was most effective for *P. aeruginosa*, other strains—*P. aeruginosa* ATCC 27853, ATCC BAA-2108, *P. aeruginosa* 01, and *P. aeruginosa* 14—were selected for testing in this experiment. In Table 9, MBIC and MBEC values for the peptides and antibiotics used alone are given, which were determined first. The results of the co-administration inhibition of biofilms by peptides and antibiotics are shown in Table 10. Heat plots of our checkerboard assays for two peptides in combination with different antibiotics against *P. aeruginosa* (FICI ≤ 0.5) are shown in Figure 7. Among the selected antibiotics, cefepime exhibited better synergistic anti-biofilm effects with peptides. Both peptides showed synergistic or additive anti-biofilm activities with cefepime against most tested *P. aeruginosa* strains.

## 3. Discussion

After Michael Zasloff discovered in 1987 that the African toad Xenopus laevis had AMP-rich glands on its skin, researchers have increasingly focused on discovering new AMPs in frogs [28]. In this study, a novel Nigrosin peptide, Nigrosin-6VL, was discovered from the skin secretions of *Odorrana andersonii*. This species is chosen as the source amphibian for AMPs mainly because they live in a complex environment, such as shadier and wetter parts of the forest in areas of high altitude and strong radiation, where they need to secrete antimicrobial substances to defend themselves against microorganisms [29]. Previous studies have demonstrated the excellent antimicrobial potential of AMPs found in the *Odorrana* species [30]. Also, peptides from the same family, such as Nigrosin-1 and Nigrosin-2 from *Rana nigromaculata*, have been found to have potent antimicrobial activity against various types of bacteria [22].

The predicted secondary structure of the origin peptide Nigrosin-6VL was an extended structure at the N-terminus with a Rana Box at the C-terminus, most likely due to the peptide’s high hydrophilic glycine (G) content, and cysteine (C) in the 15th and 21st positions forming a ring structure via a disulphide bond [25]. The secondary structure, especially the alpha-helix, is crucial for the antimicrobial functions of many AMPs as it facilitates their interaction with membranes [31]. In this study, Nigrosin-6VL was predicted to have a predominantly extended structure, which might be attributed to the presence of Gly residues in its sequence. Glycine is known to disrupt secondary structures due to its small side chain and unstable conformation [32]. This potential poor conformation could contribute to its moderate antimicrobial activities. A Rana Box with seven amino acids is the signature structure of Nigrosin family peptides. However, the impact of this structure on the Nigrosin activity remains elusive. For example, Bao et al. found that the presence of the Rana Box is not necessary for the antimicrobial activities of Nigrocin-HL, and the deletion of this structure enables the analogue Nigrocin-HLD to exhibit improved antimicrobial activities against test microorganisms [25]. Conversely, in the study by Lu et al., the preservation of the Rana Box was considered crucial for the antimicrobial functions of Nigrocin-PN [26]. Interestingly, the presence of disulphide bonds in the Rana Box may not necessarily be essential for the peptide’s antimicrobial activities. Meanwhile, previous studies have predominantly focused on examining the influence of the overall structure on peptide activity. Therefore, it remains unclear how the presence or absence of disulphide bonds specifically affects peptide activity. In this work, we designed four analogues, 1885-C, 1885-R, 1924-A, and 1782-R, by modifying the Rana Box. Specifically, we removed one or two Cys residues at positions 15 and 21 and replacing them with Ala to disrupt the formation of the disulphide bond. The antimicrobial results demonstrated that the presence of Cys residues, rather than the integrity of the disulphide bond, seems more crucial for peptide activity. This is evident from the great decrease in antimicrobial functions observed in the modified peptide 1782-R, where two Cys residues were removed. Additionally, the impact of the Cys presence on the activity against Gram-positive bacteria appears to be more pronounced than that against Gram-negative bacteria. This is highlighted by the substantial increase in GM MIC observed in the modified peptides 1885-C and 1885-R, where only one Cys residue is preserved, specifically against Gram-positive bacteria. To verify whether this inference holds true, future studies will need to conduct modification tests on more Nigrosin peptides. More charges and α-helix structures may boost the antibacterial and tumour cell growth inhibitory activity of the peptide [32,33]. Glycine is an effective N-capping agent that has been shown to impact peptides’ proclivity to form α-helices, and arginine carries a positive charge [34,35]. So, new peptides 2170-2R were finally obtained through the modification of the N-terminal of the original peptide, mainly carried out by removing Gly and adding Arg, and the antibacterial properties of the peptide were greatly boosted when compared to the origin peptide.

In a time-killing kinetics study, both peptides can rapidly limit bacterial multiplication, which is especially the case for the analogue 2170-2R, which almost eradicates bacteria upon mixing. To evaluate their antibacterial mechanisms against Gram-positive bacteria and Gram-negative bacteria, MRSA NCTC 12493 and *E. coli* ATCC 8739 were selected as representative strains for our studies. Gram-negative bacteria have both an inner and outer cytoplasmic membrane, and AMPs must first pass through the outer membrane and cell wall before hitting the inner membrane to cause cytoplasmic leakage and kill the bacteria [36]. Therefore, the NPN assay was used to test the outer membrane permeability of the peptides Nigrosin-6VL and 2170-2R against *E. coli* ATCC 8739, with the membrane-targeting peptide melittin serving as the positive control. The experimental results revealed that peptide 2170-2R exhibited a notable effect on the membrane permeability of the outer membrane of *E. coli* ATCC 8739, whereas peptide Nigrosin-6VL did not show such an effect. Then, the SYTOX green assay was used to examine peptide permeability on bacterial cytoplasmic membranes, and melittin was utilised as a positive control because it is a well-known membrane-targeting peptide that may cause pore formation in bacterial cell membranes, resulting in cytoplasmic leakage [37]. For *E. coli* ATCC 8739, the treatment of both peptides greatly increased the membrane permeability. However, for MRSA NCTC 12493, the presence of Nigrosin-6VL did not result in great changes in its membrane permeability. These results indicate that the analogue peptide 2170-2R may exert its antibacterial functions by rapidly inducing membrane permeability changes, causing leakage of the bacterial cellular contents and thereby leading to death. Meanwhile, for Nigrosin-6VL, the mechanism might be different. For *E. coli* ATCC 8739, it may penetrate the outer membrane and peptidoglycan layer via specific routes, subsequently targeting the inner membrane for bacterial destruction. Meanwhile, for MRSA NCTC 12493, the presence of Nigrosin-6VL cannot induce great changes in the cytoplasmic membrane as it did on tested *E. coli* cells, indicating that its target may not be the membrane. To validate its precise mechanism, more experiments need to be carried out.

Given the membrane-disruptive effects of peptides, four conventional antibiotics with different antimicrobial mechanisms were selected for synergistic antibacterial experiments with the peptides Nigrosin-6VL and 2170-2R. According to the results of co-administration assays, synergistic or additive effects of the two peptides generally appeared when they acted together with cefepime, levofloxacin, and gentamicin. The inhibitory effects of the two peptides with cefepime on *K. pneumoniae* ATCC 43816 and *E. faecalis* NCTC 12697, and the peptides with gentamicin on *K. pneumoniae* ATCC 43816 and *E. coli* ATCC 8739, were the most potent of all groups. Following synergy, the MIC values of peptides and antibiotics were both decreased to roughly one-sixteenth of the values when used alone. Gentamicin is widely used in clinical treatment due to its strong antibacterial activity and broad-spectrum action, while nephrotoxicity and ototoxicity are significant problems associated with the usage of aminoglycosides [28,38,39]. Meanwhile, the broad-spectrum cephalosporin cefepime remains an essential medication against severe bacterial infections; however, it may be associated with higher fatality rates when compared to other β-lactam antibiotics [40]. So, co-administration of gentamicin or cefepime with peptides can lower medication concentrations, potentially reducing harmful side effects and drug resistance, which may provide suitable conditions for future studies on the clinical application of pharmaceuticals. In a different finding, the synergistic antibacterial activity of the two peptides and vancomycin was not clear, and antagonistic results were seen. These findings may be related to the distinct peptides and antibiotics’ bactericidal mechanisms. The main antibacterial site of gentamicin is the ribosome, as one of the aminoglycoside antibiotics, and DNA gyrase is a target of the quinolone levofloxacin [41,42]. When the peptides with a permeability function were combined with two antibiotics, this could make it easier for antibiotics to enter the bacterial cytoplasm, allowing medicines to exhibit a greater antibacterial impact. Meanwhile, the results of antibacterial synergy also showed that the antibacterial non-membrane targets of the two peptides may differ from gentamicin and levofloxacin; otherwise, they may affect the antibacterial activity by competing for the same target. Vancomycin works by attaching itself to the terminal d-Ala-d-Ala moiety of uncrosslinked Lipid II. This prevents penicillin-binding proteins (PBPs) from crosslinking Lipid II into mature peptidoglycan, which weakens the cell envelope and increases the risk of osmotic stress and cell rupture [43,44]. However, cefepime, as a β-lactam antibiotic, can also kill bacteria by attaching to PBPs and inhibiting the formation of cell walls. The two antibiotics’ methods of action were identical, but the results of synergistic antibacterial actions with peptides were radically different. This finding might be because the glycopeptide antibiotic vancomycin and the peptides used share the same non-membrane antibacterial target and the interaction between the peptides and Lipid II altered vancomycin’s typical antibacterial activity.

*P. aeruginosa* is a common nosocomial pathogen that causes a broad range of acute and chronic infections and is subject to antibiotic resistance to many kinds of traditional antibiotics, such as aminoglycosides, quinolones, and β-lactams, which cause common antimicrobial treatments such antibiotics to typically show low efficiency, increasing mortality [45,46,47]. From previous clinical studies, it is established that *P. aeruginosa* is the most common pathogen responsible for cystic fibrosis (CF) lung infections. Persistent infections with this bacterium often develop resistance to antibiotic therapy, leading to worsened lung function and ultimately contributing to the mortality of cystic fibrosis patients [12]. Furthermore, *P. aeruginosa* has been linked to a higher death rate in patients with chronic obstructive pulmonary disease, accounting for more than 5% of infectious exacerbations in this population [48]. The World Health Organization (WHO) has recently listed carbapenem-resistant *P. aeruginosa* as one of three bacterial species in which there is a critical need for the development of new antibiotics to treat infections [49]. The biofilm of *P. aeruginosa* has the potential to induce multiple forms of infection in individuals who have indwelling inert surfaces, such as internal or external medical devices [45,50]. The biofilms of *P. aeruginosa*, which shield the bacterium from external stressors and prevent phagocytosis, confer colonisation and long-term survival, making treatment of these infections more difficult [51]. *P. aeruginosa* develops an adaptive resistance through the production of biofilm in the lungs of infected individuals, which acts as a diffusion barrier to prevent antibiotics from reaching the bacterial cells [52]. Furthermore, biofilm may develop multidrug-tolerant persister cells that can withstand antibiotic treatment; these cells are the cause of persistent and recurring infections in people with cystic fibrosis [53]. Treatment of biofilm-associated infections is very challenging because bacteria in biofilms become increasingly resistant to the effects of antibiotics and the human immune system [45,50]. More effective and safer drugs are therefore needed to treat infections of *P. aeruginosa*. Many studies have proved that AMPs can be employed to prevent biofilm growth because of their several promising properties [54]. Since peptide–antibiotic combinations were more effective in inhibiting biofilms of common *P. aeruginosa* in this study, they were tested for their activity against clinically isolated and drug-resistant *P. aeruginosa* from different sources. Similar to the results for the biofilm of *P. aeruginosa* ATCC CRM 9027, subsequent use of peptides in synergy with antibiotics reduced the concentration needed to inhibit biofilms of drug-resistant bacteria. The most promising outcomes were achieved by combining two peptides with gentamicin against *P. aeruginosa* ATCC 27853 and Nigrosin-6VL against *P. aeruginosa* 14, which reduced the effective concentration of peptides and antibiotics to one-eighth of the original level. These results may be attributed to the membrane permeability mechanism of the peptide aiding the antibiotics in entering the planktonic bacteria, thereby affecting the formation of the biofilm. Additionally, peptides and antibiotics may exert an inhibitory effect on the biofilm system, such as disrupting information exchange.

## 4. Materials and Methods

### 4.1. Acquisition of Skin Secretions from Odorrana andersonii

Electrical stimulation with 5 V, 100 Hz, and 140 ms width was used to extract the dorsal skin secretions from adult *Odorrana andersonii* specimens, as repeated at 20 s intervals for 2 min. The skin secretions were washed off the skin with deionised water and stored at −20 °C before being snap-frozen in liquid nitrogen and lyophilised. This study was carried out under the UK Animal (Scientific Procedures) Act 1986, project license PPL 2694, issued by the Northern Ireland Department of Health, Social Services, and Public Safety. The Institutional Animal Care and Use Committee (IACUC) at Queen’s University Belfast approved the procedures on 1 March 2011.

### 4.2. Molecular Cloning

Dynabeads oligo(dT)25 (Invitrogen^TM^, Oslo, Norway) was used to isolate pure mRNA from the skin secretion of *Odorrana andersonii* since it could bind polyadenylated mRNA in the cell lysis solution provided with the kit. With a SMART-RACE kit (Takara Bio, Kusatsu, Japan), reverse transcription and first-strand cDNA synthesis were followed by a 3’-RACE procedure-specific primer to isolate target antimicrobial peptide precursor nucleic acid sequence data. The 3′-RACE reaction applied a nested universal primer (NUP, provided in the kit) and a degenerate sense primer (S1:5′-GGCTTYCCTGAAGAAATCTC-3′, Y = C + T) designed according to an N-terminal sequence—AS/FLKKS—of the highly conserved signal peptide of neobatrachian frog skin AMP precursors [55]. The PCR cycling protocol includes an initial denaturation phase at 94 °C for 60 s, followed by 40 thermal cycles of denaturation at 94 °C for 30 s, primer annealing for 30 s at 60 °C, and extension at 72 °C for 180 s. Gel electrophoresis was used to purify the PCR results, which were then cloned using a pGEM-T vector system (Promega, Madison, WI, USA). The 3’-RACE reactions were then purified, cloned, and sequenced in the right order using a Rapid PCR purification system (E.Z.N.A. Cycle Pure Kit (V-spin), Omega Bio-Tek D6492-02, Norcross, GA, USA) and an ABI 3100 automated sequencer (Applied Biosystems, Foster City, CA, USA).

### 4.3. Peptide Sequence Analysis, Secondary Structural Prediction, and Verification

BLAST (https://blast.ncbi.nlm.nih.gov/Blast.cgi) was used for the entire amino acid sequence of the precursor to hunt for a similar sequence, and then Omega Clustal (https://www.ebi.ac.uk/Tools/msa/clustalo/) (accessed on 3 June 2024) was used to confirm the highly conserved domain. To predict the physicochemical properties of all peptides, the Antimicrobial Peptide Calculator and Predictor of the APD3 database (https://aps.unmc.edu/) (accessed on 3 June 2024) was utilised, while the possible secondary structure of Nigrosin-6VL was predicted using the PEPFOLD3 website (https://bioserv.rpbs.univ-paris-diderot.fr/services/PEP-FOLD3/) (accessed on 3 June 2024).

### 4.4. Synthesis and Purification of Peptides

**Figure 8:**
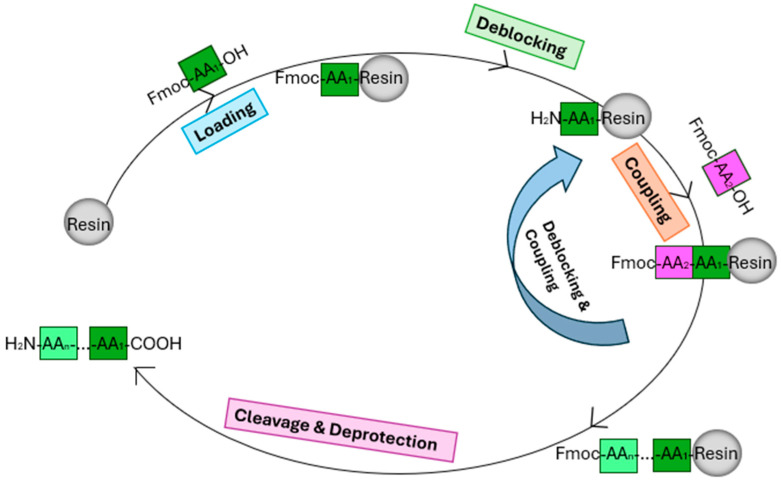
Scheme of solid-phase peptide synthesis (SPPS) steps.

Vials containing 0.75 mol of amino acids and 2-(1*H*-Benzotriazole-1-yl)-1,1,3,3-tetramethyluronnium hexafluorophosphate (HBTU) were prepared, while 0.3 mol of Fmoc-Cys (Trt)-Wang resin (Novabiochem, London, UK) was placed in a separate reaction vessel prior to synthesis. The antimicrobial peptides (AMPs) were synthesised using solid-phase peptide synthesis (Tribute 2-channel peptide synthesiser, Protein Technologies, Tucson, AZ, USA) [56]. For cleavage, a mixture solution (25 mL per 1 g resin) was prepared as follows: 94% trifluoroacetic acid (TFA), 2% H_2_O, 2% thioanisole (TIS), and 2% 1,2-ethanedithiol (EDT), stirred at room temperature for 2 h. The scheme of the SPPS is shown in Figure 8. The peptide was dissolved in buffer A, consisting of 99.5% ddH_2_O and 0.05%TFA, along with 10% of the total volume of dimethyl sulfoxide (DMSO). This mixture was allowed to react overnight to facilitate the formation of disulphide bonds before purification using RP-HPLC (Waters, Cheshire, UK) [57]. The purity and authenticity were determined by MALDI-TOF MS (4800 MALDI-TOF/TOF, Applied Biosystems, Foster City, CA, USA).

### 4.5. Anti-Planktonic Microorganism Activity Study

The minimum inhibitory concentration (MIC) is the minimum concentration of an antimicrobial agent that prevents the visible growth of a bacterium or bacteria. The MIC is an important piece of data for research on new antimicrobial agents and helps in the effective selection of antibiotics to improve the success of treatment. The minimum bactericidal concentration (MBC) is defined as the lowest antibiotic concentration that kills 99.9% of the inoculum. This study mainly contained eight different microorganisms, which were *Staphylococcus aureus* (ATCC 6538), methicillin-resistant *Staphylococcus aureus* (NCTC 12493), Enterococcus faecalis (NCTC 12697), *Escherichia coli* (ATCC 8739), *Klebsiella pneumoniae* (ATCC 43816), *Pseudomonas aeruginosa* (ATCC CRM 9027), *Acinetobacter baumannii* (BAA 747), and *Candida albicans* (ATCC 10231). The bacteria were incubated in the compatible medium at 37 °C overnight.

### 4.6. Haemolysis Assay and TI Value Calculation

The pure peptide was dissolved in PBS and then two-fold diluted to obtain a series of concentrations: 512, 256, 128, 64, 32, 16, 8, 4, and 2 μM. A positive control (0.1% Triton) and blank control (PBS) were used. Two millilitres of fresh horse red blood cells were washed in PBS until the supernatant became colourless and clear, and the mixture was then thoroughly mixed. Each mixture contained 100 μL of peptide solution or controls and 100 μL of horse red blood cells, which were then incubated at 37 °C for 2 h and centrifuged at 930× *g* for 10 min. Subsequently, 100 μL of supernatant from each tube was transferred into a 96-well plate, and the plate reader measured the absorbance at a wavelength of 570 nm. The haemolysis rate was calculated using the following formula:

Percentage Haemolysis= (A_Sample_ − A_Blank_)/(A_Positive_ − A_Blank_) × 100%

The therapeutic potential of antimicrobial agents was valued based on their selectivity toward bacteria in reference to erythrocytes. The TI value was calculated from the results of antibacterial and haemolysis experiments of peptides using the following formula:

TI value = HC10 / GM_MICs_.

GM was the geometric mean of MICs against all tested bacteria.

### 4.7. Time-Killing Kinetic Assay

The bacteria were subcultured as described previously to perform the MIC assay. Bacterial inoculation was carried out using peptide concentrations equivalent to 4× MIC, 2× MIC, and MIC, with a bacterial concentration of 5 × 10^5^ CFU/mL. After adding the peptides and DMSO to the bacterial suspensions, all groups were mixed and then incubated at 37 °C. Samples and controls were collected at different time points (0, 5, 10, 20, 30, 60, 90, and 120 min), and the mixture was transferred to sterile PBS, diluted tenfold, and then further diluted four times. Each dilution of bacteria/peptide samples at different concentrations was plated onto the appropriate agar plate for live cell counting. After incubation at 37 °C for 24 h, the number of colonies at different concentrations and time points was determined. Finally, the number of bacteria at different time points was plotted as a line graph to illustrate the antibacterial kinetics of the peptides.

### 4.8. The SYTOX Green of the Bacterial Membrane Permeabilisation Assay

To confirm the permeability mechanisms of the peptides Nigrosin-6VL and 2170-2R, *E. coli* ATCC 8739 and MRSA NCTC 12493 were, respectively, incubated with the peptides (1×, 2×, and 4× MIC) in the presence of SYTOX Green dye (Life Technologies, Renfrew, UK) and the fluorescence intensity was monitored. These two bacteria were inoculated in a TSB medium and grown to the logarithmic phase. The supernatant was decanted after centrifugation (1000× *g*, 10 min, 4 °C). The bacteria were washed with 5% TSB in 0.85% NaCl solution and resuspended until a 1 × 10^8^ CFU/mL density was reached, and the solution was then assessed by measuring the OD value (0.7) at wavelength 590 nm. We added 40 µL of two peptides to a black 96-well plate. Meanwhile, 10 µL of diluted SYTOX green-fluorescent nucleic acid stain was transferred into each well. Next, 50 µL of the bacterial suspension (1 × 10^8^ CFU/mL) was transferred into each well. The plate was analysed by the Synergy HT plate reader (Bio-Tek, Winooski, VT, USA) using a 2 h kinetic programme with excitation at 485 nm and emission at 580 nm.

### 4.9. Outer Membrane Assay

This study involved an outer membrane permeability assay using *N*-Phenyl-1-naphthylamine (NPN), a fluorescent dye known for its interaction with the outer membrane of Gram-negative bacteria. Initially, *E. coli* ATCC 8739 was cultured in LB medium and subjected to overnight incubation at 37 °C. Subsequently, the cultures were subcultured at 37 °C with agitation at 120 rpm for 2 h. The cells were then centrifuged at 2000 rpm for 10 min, and the resulting cell pellets were washed and diluted to an optical density (OD) of 0.50 at a wavelength of 600 nm, corresponding to a concentration of 1 × 10^8^ CFU/mL. This dilution was achieved using a 5 mM HEPES buffer solution supplemented with 5 mM glucose, adjusted to a pH of 7.4. The bacterial solution was further diluted to a concentration of 1 × 10^7^ CFU/mL. Subsequently, 100 μL of bacterial culture was combined with 50 μL of peptide solution in a black 96-well plate. Peptide concentrations were determined based on MIC values obtained from assays targeting planktonic microorganisms. Growth control was established using the HEPES buffer. Then, 50 μL of NPN (at a final concentration of 10 μM per well) was added to the respective wells. Melittin at a concentration of 16 µM was used as the positive control in the experiment [58]. Real-time fluorescence measurements were conducted using a Synergy HT plate reader (BioTek, Washington, DC, USA) with excitation and emission wavelengths set at λ = 360 nm and λ = 460 nm, respectively, for a duration of 60 min. The experiment was performed in triplicate, with each trial conducted independently.

### 4.10. Anti-Biofilm Assay

The minimum bactericidal concentration (MBC) is defined as the lowest antibiotic concentration that kills 99.9% of the inoculum. The minimum biofilm inhibitory concentration (MBIC) is the lowest concentration that inhibits at least 90% of the formation of biofilm. The minimum biofilm eradication concentration (MBEC) is the minimum concentration that can eradicate at least 99% of the biofilm. In addition to the eight microorganisms described previously, this experiment was also applied to *P. aeruginosa* (ATCC 27853, ATCC BAA-2108, *P. aeruginosa* 01, *P. aeruginosa* 14).

### 4.11. Peptide and Antibiotic Co-Administration Study

#### 4.11.1. Peptide and Antibiotic Co-Administration of MIC Assay

The bacteria were cultured, subcultured, diluted, and incubated as the methods of the MIC assay. The pure peptides and antibiotics were weighed and, respectively, dissolved in DMSO and dd water to make the final concentration of 2× MIC. Then, the solutions were double diluted to achieve a series of concentrations from 2× MIC to 0.125× MIC. Different concentrations of peptide and antibiotic solutions were added to a 96-well plate in the form of a checkerboard grid. The blank control and growth control were still 100 µL medium and 100 µL bacterial culture. The plate was incubated at 37 °C for 24 h. After that, the plate reader was used to monitor the absorbance of each well at 550 nm (The Synergy TM HT, BioTek, Winooski, VT, USA).

Parameter fractional inhibitory concentration indices (FICIs) were used to evaluate the synergistic effect, and the value of that was calculated as follows:

FICI = FICa + FICb

FICa = MIC combination peptide/MIC single peptide

FICb = MIC combination antibiotic/MIC single antibiotic

The FICI value was interpreted as “synergy” (FICI ≤ 0.5), “no interaction” (0.5 < FICI ≤  4), or “antagonism” (FICI > 4).

#### 4.11.2. Peptide and Antibiotic Co-Administration of MBIC Assay

The bacteria were also cultured, subcultured, diluted, and incubated as the methods of the MBIC assay. All experimental groups were included in a peptide and antibiotic co-administration of MIC assay. To ensure that the mixture was evenly dispersed, the plate was placed in a shaker and incubated for 24 h at 37 and 220× *g*. The plate was twice rinsed with 200 µL of PBS solution following a 24 h incubation period with peptides and diluted bacterial cultures. Each well received a separate addition of 100 μL of methanol and 0.1% crystal violet solution (Sigma-Aldrich, London, UK), which was left there for 20 min. The plate was then carefully cleaned under running water. Following the drying process, each well received 160 µL of acetic acid (33% in dd water, Sigma-Aldrich, UK). Additionally, the absorbance was measured at λ = 595 nm using the Synergy HT plate reader. FICI values were calculated and identified for our peptide and antibiotic co-administration of MIC assay.

### 4.12. Method of Measurement and Statistical Analysis

All data for these bioactive assays were obtained through nine repeats of three independent experiments. Negative, positive, and growth controls were represented by letters of the alphabet on the icon’s abscissa, such as “N,” “P,” and “G”. The software GraphPad Prism 6 was used to calculate the HC50 and IC50 values (GraphPad Software, La Jolla, CA, USA).

## 5. Conclusions

In summary, Nigrosin-6VL was identified as a novel peptide found in the frog skin secretion of *Odorrana andersonii*, while the peptide 2170-2R was obtained through peptide modification. When used in combination with conventional antibiotics, both peptides demonstrated the potential to enhance antibacterial activity while reducing the effective concentration needed. Furthermore, they exhibited the ability to prevent bacterial biofilm formation, possibly due to their mechanism of bacterial membrane permeability.

## Figures and Tables

**Table 1:** Peptide sequences’ calculated molecular mass and net charge for all peptides.

Peptide	Sequence	Calculated Molecular Mass	Charge
Nigrosin-6VL	GLLSGVLGAGKKIVCGLSGRC	1986.088	+3
1885-C	GLLSGVLGAGKKIV-GLSGRC	1885.099	+3
1885-R	GLLSGVLGAGKKIVCGLSGR-	1885.547	+3
1924-A	GLLSGVLGAGKKIVAGLSGRA	1924.037	+3
1782-R	GLLSGVLGAGKKIV-GLSGR-	1782.097	+3
2057-C	GLLRGVLGAGKKIVCGLSGRC	2057.665	+4
2085-R	GLLRAVLAAGKKIVCGLSGRC	2084.890	+4
2170-2R	GLLRAVLRAGKKIVCGLSGRC	2169.932	+5

Mutation sites are labelled yellow.

**Table 2:** MIC and MBC values (µM) of Nigrosin-6VL and its analogues.

Strains (Codes)	MICs/MBCs (µM)							
	Nigrosin-6VL	1782-R	1885-C	1924-A	1885-R	2057-C	2085-R	2170-2R
*S. aureus* (ATCC 6538)	16/32	>128/>128	>128/>128	128/>128	128/>128	128/>128	16/16	4/8
MRSA (NCTC 12493)	>128/>128	>128/>128	>128/>128	>128/>128	64/64	64/128	16/32	16/16
*E. faecalis* (NCTC 12697)	>128/>128	>128/>128	>128/>128	>128/>128	>128/>128	>128/>128	64/256	128/128
*E. coli* (ATCC 8739)	64/128	128/>128	16/32	64/128	32/64	16/32	16/16	4/8
*K. pneumoniae* (ATCC 43816)	128/128	>128/>128	64/128	64/128	32/64	64/128	32/64	32/32
*P. aeruginosa* (ATCC CRM 9027)	32/128	>128/>128	64/128	128/>128	32/64	64/128	64/64	32/32
*A. baumannii* (BAA 747)	>128/>128	>128/>128	>128/>128	>128/>128	128/>128	>128/>128	32/64	4/8
*C. albicans* (ATCC 10231)	128/>128	>128/>128	>128/>128	64/128	>128/>128	>128/>128	32/32	64/128

**Table 3:** HC10 and TI values of peptides.

Peptide	HC10 (μM)	GM (μM)			TI Value *		
		G+ Bacteria	G− Bacteria	Yeast	G+ Bacteria	G− Bacteria	Yeast
Nigrosin-6VL	>256	101.59	90.51	128.00	5.04	5.66	4.00
1782-R	>256	256.00	215.27	256.00	2.00	2.38	2.00
1885-C	>256	256.00	64.00	256.00	2.00	8.00	2.00
1924-A	>256	203.19	107.63	64.00	2.52	4.76	8.00
1885-R	>256	128.00	45.25	256.00	4.00	11.31	2.00
2057-C	>256	128.00	64.00	256.00	4.00	8.00	2.00
2085-R	>256	25.40	32.00	32.00	20.16	16.00	16.00
2170-2R	>256	25.16	11.31	64.00	20.35	45.27	8.00

* TI value was calculated as HC10/GM. Since HC10 values are higher than 256 μM, 512 μM was used to calculate the TI value.

**Table 4:** MIC and MBC values (µM) of antibiotics.

Strains	MICs/MBCs (µM)			
	Levofloxacin	Cefepime	Gentamicin	Vancomycin
*S. aureus* ATCC 6538	0.5/0.5	32/64	2/2	8/64
MRSA NCTC 12493	0.25/0.5	16/32	1/2	4/8
*E. faecalis* NCTC 12697	2/8	128/>128	16/32	32/>128
*E. coli* ATCC 8739	0.25/0.5	8/16	2/4	32/>128
*K. pneumoniae* ATCC 43816	>128/>128	128/>128	1/1	>128/>128
*P. aeruginosa* ATCC CRM 9027	2/2	2/2	0.25/0.5	>128/>128
*A. baumannii* BAA 747	1/1	1/2	>128/>128	>128/>128

**Table 5:** FICI values of peptide and antibiotic co-administration.

Strains	FICI Values							
	Vancomycin		Cefepime		Levofloxacin		Gentamicin	
	Nigrosin-6VL	2170-2R	Nigrosin-6VL	2170-2R	Nigrosin-6VL	2170-2R	Nigrosin-6VL	2170-2R
*S. aureus* ATCC 6538	2.25	0.625	0.625	1	0.625	0.625	1.5	1
MRSA NCTC 12493	1	0.625	0.75	0.75	2	1.125	0.5	0.375
*E. faecalis* NCTC 12697	0.625	0.625	0.125	0.15625	0.625	0.625	0.625	0.625
*E. coli* ATCC 8739	1.125	>4	0.375	0.53125	0.375	0.375	0.125	0.125
*K. pneumoniae* ATCC 43816	1.125	1.125	0.1875	0.1875	0.625	1	0.1875	0.15625
*P. aeruginosa* ATCC CRM 9027	>4	1.125	0.5	0.5	0.5	0.625	0.5	0.5
*A. baumannii* BAA 747	1.25	0.625	1	1.5	>4	>4	0.1875	0.25

**Table 6:** MBICs and MBECs (µM) of two peptides.

Strains	MBICs/MBECs (µM)	
	Nigrosin-6VL	2170-2R
*S. aureus* ATCC 6538	128/>128	4/16
MRSA NCTC 12493	>128/>128	4/16
*E. faecalis* NCTC 12697	>128/>128	32/>128
*K. pneumoniae* ATCC 43816	>128/>128	32/>128
*P. aeruginosa* ATCC CRM 9027	>128/>128	>128/>128
*A. baumannii* BAA 747	>128/>128	8/16

**Table 7:** MBICs and MBECs (µM) of antibiotics.

Strains	MBICs/MBECs (µM)	
	Cefepime	Gentamicin
*S. aureus* ATCC 6538	4/8	8/16
MRSA NCTC 12493	>128/>128	2/>128
*E. faecalis* NCTC 12697	>128/>128	>128/>128
*K. pneumoniae* ATCC 43816	>128/>128	32/32
*P. aeruginosa* ATCC CRM 9027	>128/>128	2/4
*A. baumannii* BAA 747	128/>128	8/16

**Table 8:** FICI values of peptide and antibiotic anti-biofilm co-administration.

Strains	Biofilm FICI Values			
	Cefepime		Gentamicin	
	Nigrosin-6VL	2170-2R	Nigrosin-6VL	2170-2R
*S. aureus* ATCC 6538	1	1	0.5	0.25
MRSA NCTC 12493	0.75	0.5	0.375	1.125
*E. faecalis* NCTC 12697	0.5	0.375	0.5	0.6125
*K. pneumoniae* ATCC 43816	1.25	3	0.5	1.125
*P. aeruginosa* ATCC CRM 9027	0.3125	0.5	0.5	1
*A. baumannii* BAA 747	0.5	0.625	0.5	0.75

**Table 9:** MBICs and MBECs (µM) of antibiotics and peptides against *P. aeruginosa* strains.

Strains	MBICs/MBECs (µM)			
	Cefepime	Gentamicin	Nigrosin-6VL	2170-2R
*P. aeruginosa* ATCC CRM 9027	>128/>128	2/4	>128/>128	>128/>128
*P. aeruginosa* ATCC 27853	2/4	4/>128	>128/>128	8/16
*P. aeruginosa* ATCC BAA-2108	64/>128	16/128	>128/>128	>128/>128
*P. aeruginosa* 01	4/>128	4/8	>128/>128	32/64
*P. aeruginosa* 14	8/>128	2/4	>128/>128	>128/>128

**Table 10:** FICI values of peptide and antibiotic anti-biofilm co-administration against *P. aeruginosa* strains.

Strains	Biofilm FICI Values			
	Cefepime		Gentamicin	
	Nigrosin-6VL	2170-2R	Nigrosin-6VL	2170-2R
*P. aeruginosa* ATCC CRM 9027	0.3125	0.5	0.5	1
*P. aeruginosa* ATCC 27853	1	0.5	0.25	0.25
*P. aeruginosa* ATCC BAA-2108	0.625	1	1	>3
*P. aeruginosa* 01	0.625	0.5	0.625	1
*P. aeruginosa* 14	0.25	0.375	0.5	1

## Data Availability

The data that support the findings of this study are available from the corresponding author upon reasonable request.

## Supplementary Materials

The following supporting information can be downloaded at https://www.mdpi.com/article/10.3390/antibiotics13070574/s1, Figure S1. MALDI-TOF MS spectrum and purity of Nigrosin-6VL and its analogues.
