# Supplementary material for: Modification and Synergistic Studies of a Novel Frog Antimicrobial Peptide against Pseudomonas aeruginosa Biofilms
Source: Antibiotics (Basel). 2024 Jun 21;13(7):574. doi: 10.3390/antibiotics13070574 (PMC11274128; doi:10.3390/antibiotics13070574)
Supplement: Supplementary file 1 [file antibiotics-13-00574-s001.zip › antibiotics-3065463-supplementary.pdf]

Nigrosin-6VL

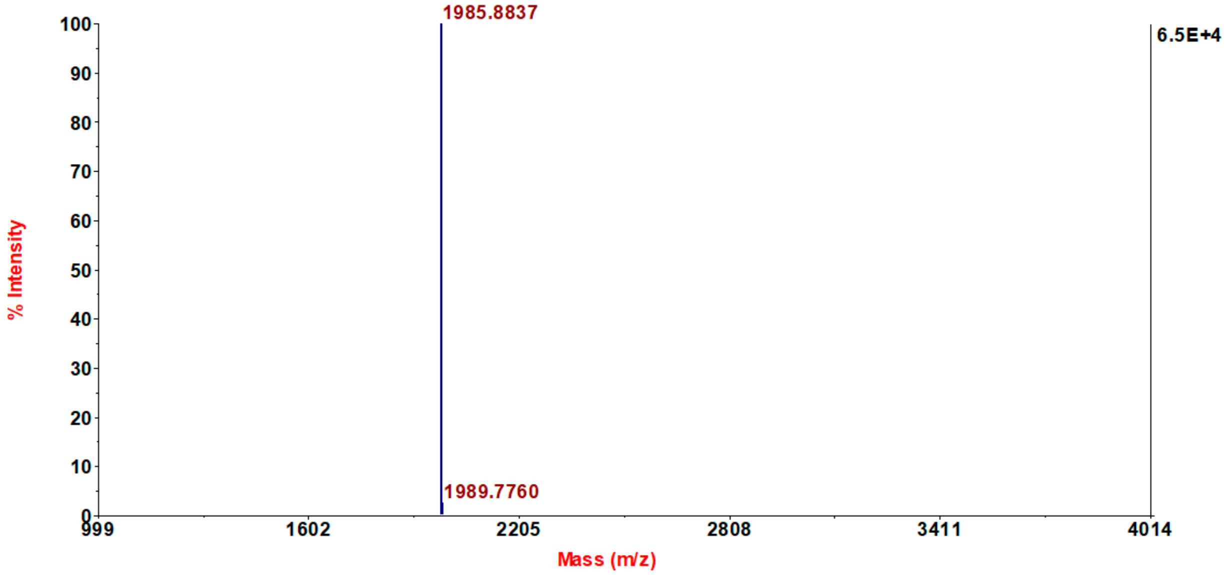

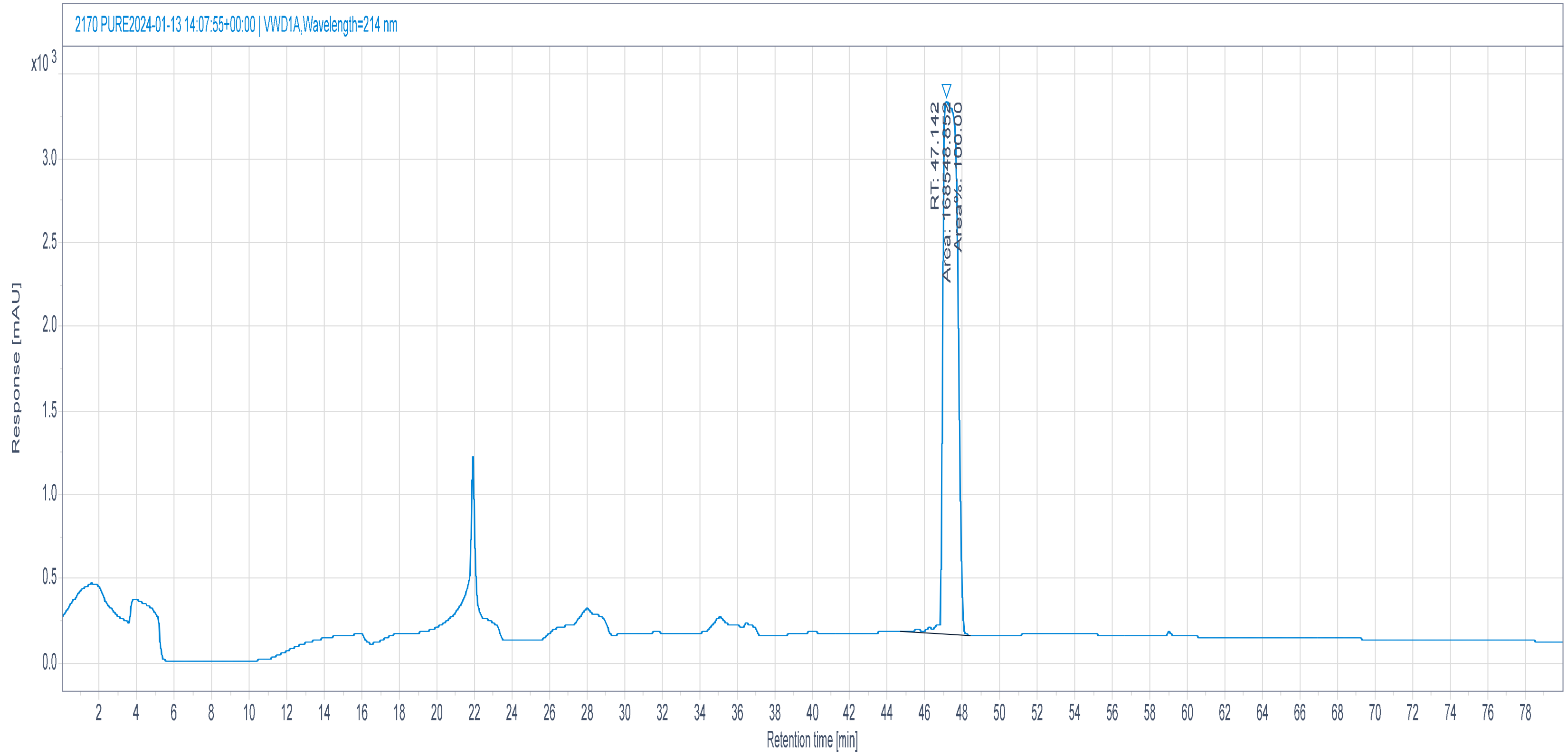
2170 PURE2024-01-13 14:07:55+00:00 | VWD1A,Wavelength=214 nm
x10 3
0.0
0.5
1.0
1.5
2.0
2.5
3.0
Response [mAU]
2
4
6
8
10
12
14
16
18
20
22
24
26
28
30
32
34
36
38
40
42
44
46
48
50
52
54
56
58
60
62
64
66
68
70
72
74
76
78
Retention time [min]
RT: 47.142
Area: 168548.852
Area%: 100.00

Pure 1782-R

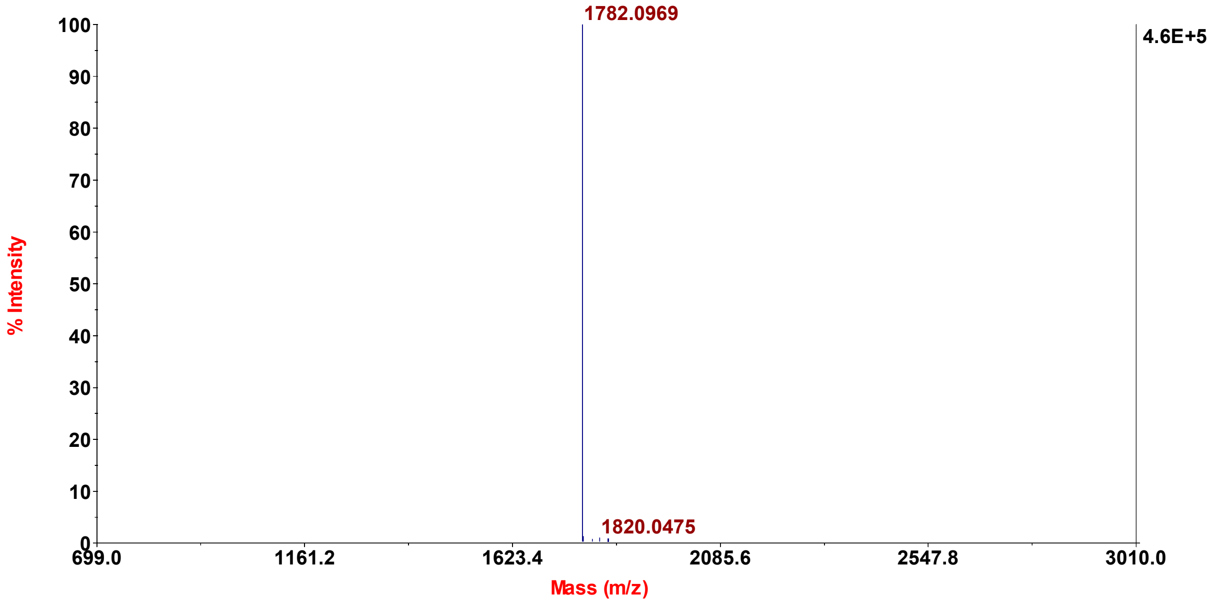

1782.0969
1820.0475
4.6E+5
% Intensity
100
90
80
70
60
50
40
30
20
10
0
699.0
1161.2
1623.4
2085.6
2547.8
3010.0
Mass (m/z)

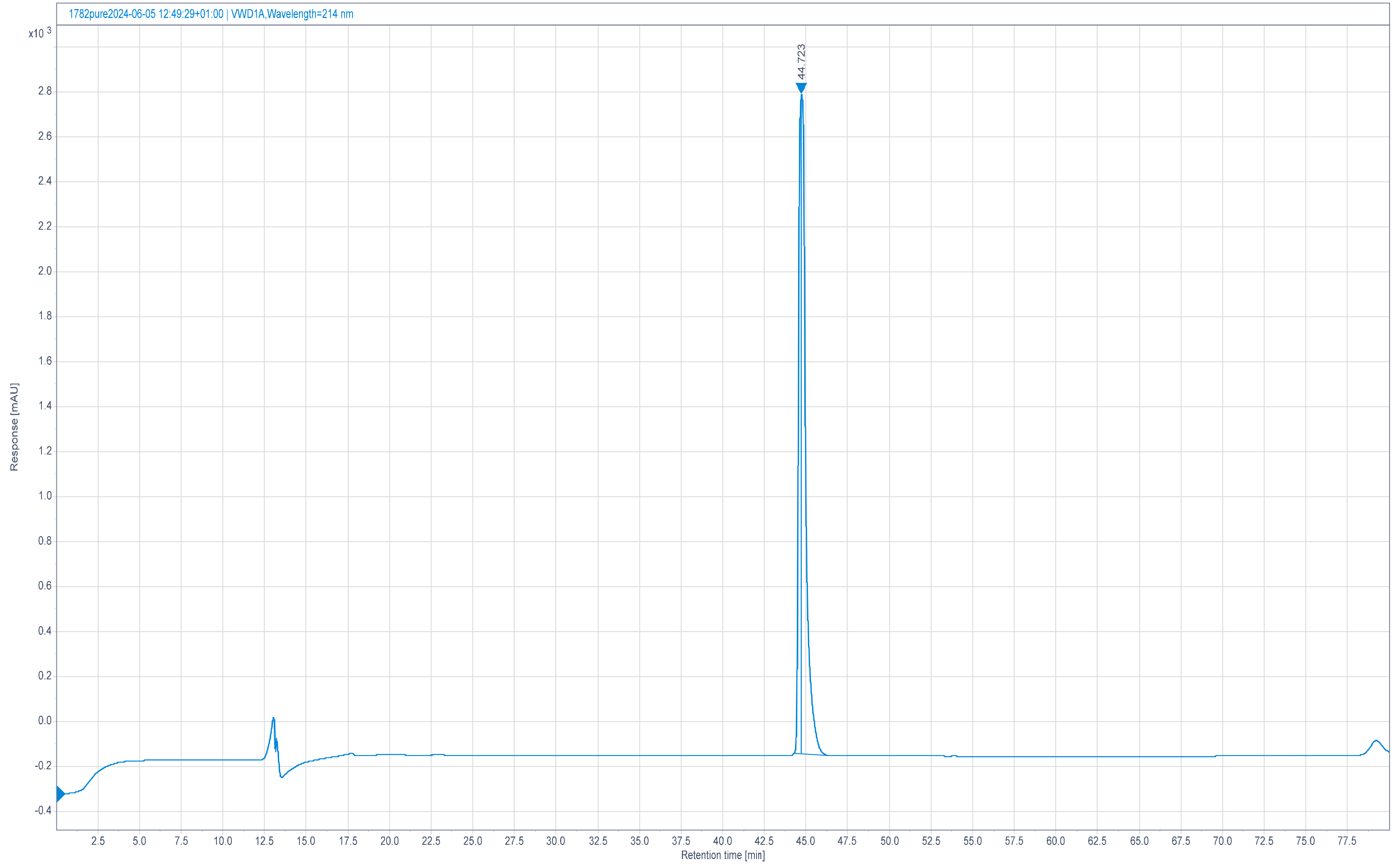
1782pure2024-06-05 12:49:29+01:00 | VWD1A,Wavelength=214 nm
x10 3
Response [mAU]
-0.4
-0.2
0.0
0.2
0.4
0.6
0.8
1.0
1.2
1.4
1.6
1.8
2.0
2.2
2.4
2.6
2.8
44.723
2.5
5.0
7.5
10.0
12.5
15.0
17.5
20.0
22.5
25.0
27.5
30.0
32.5
35.0
37.5
40.0
42.5
45.0
47.5
50.0
52.5
55.0
57.5
60.0
62.5
65.0
67.5
70.0
72.5
75.0
77.5
Retention time [min]

1885c

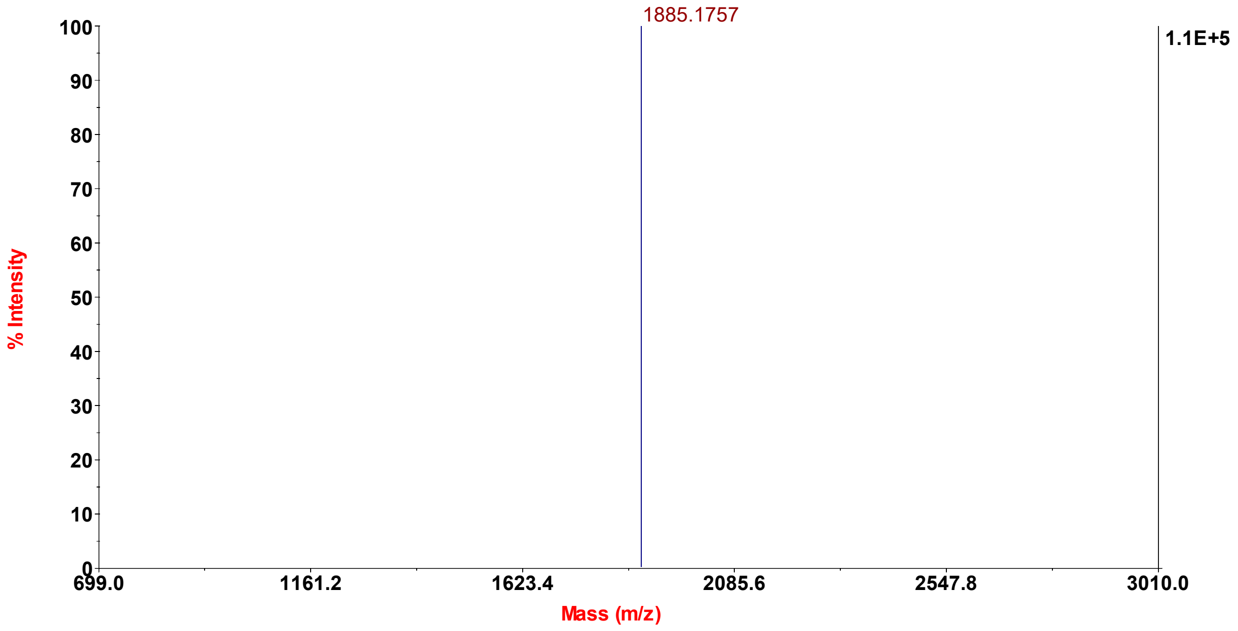
1885.1757
1.1E+5
% Intensity
100
90
80
70
60
50
40
30
20
10
0
699.0
1161.2
1623.4
2085.6
2547.8
3010.0
Mass (m/z)

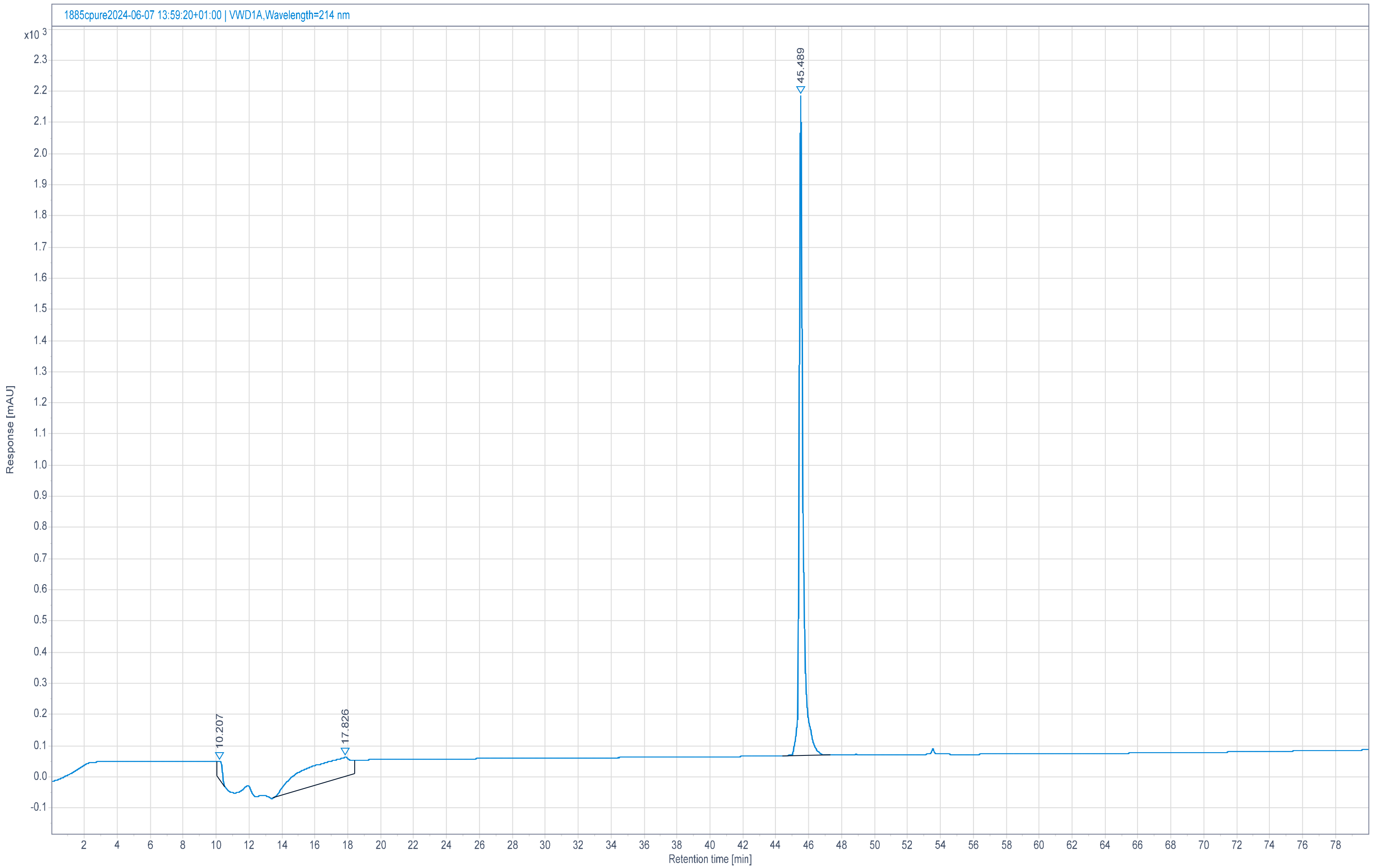

1885cpure2024-06-07 13:59:20+01:00 | VWD1A,Wavelength=214 nm
x10 3
Response [mAU]
Retention time [min]
10.207
17.826
45.489

1885r

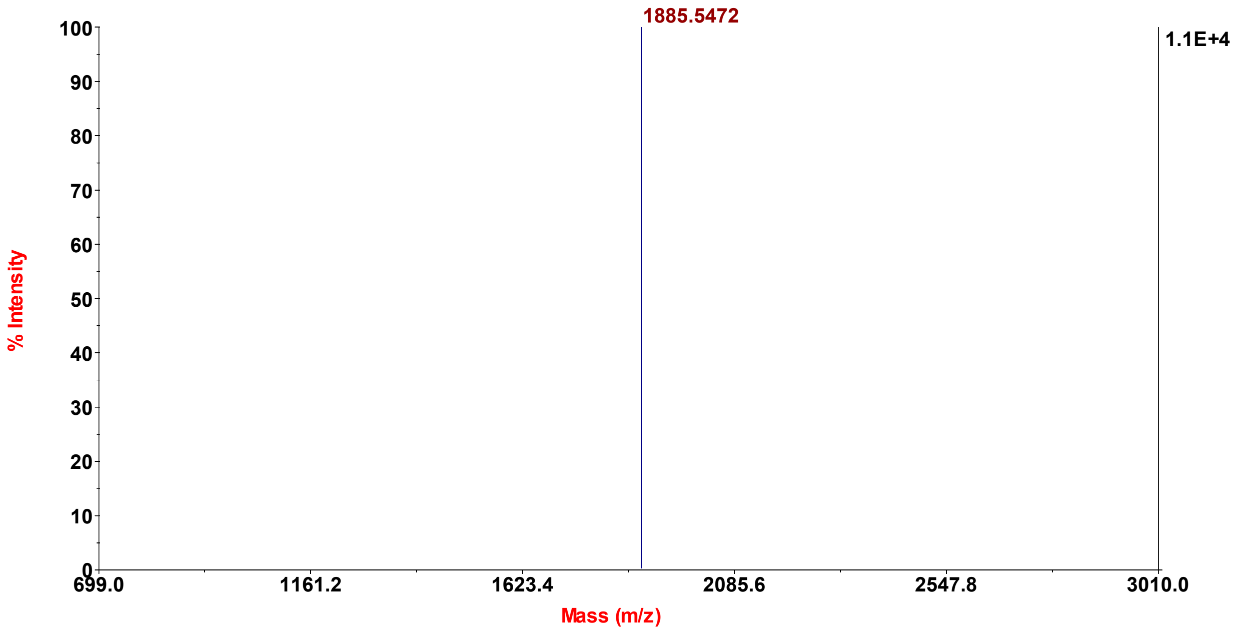
1885.5472
1.1E+4
100
90
80
70
60
50
40
30
20
10
0
% Intensity
699.0
1161.2
1623.4
2085.6
2547.8
3010.0
Mass (m/z)

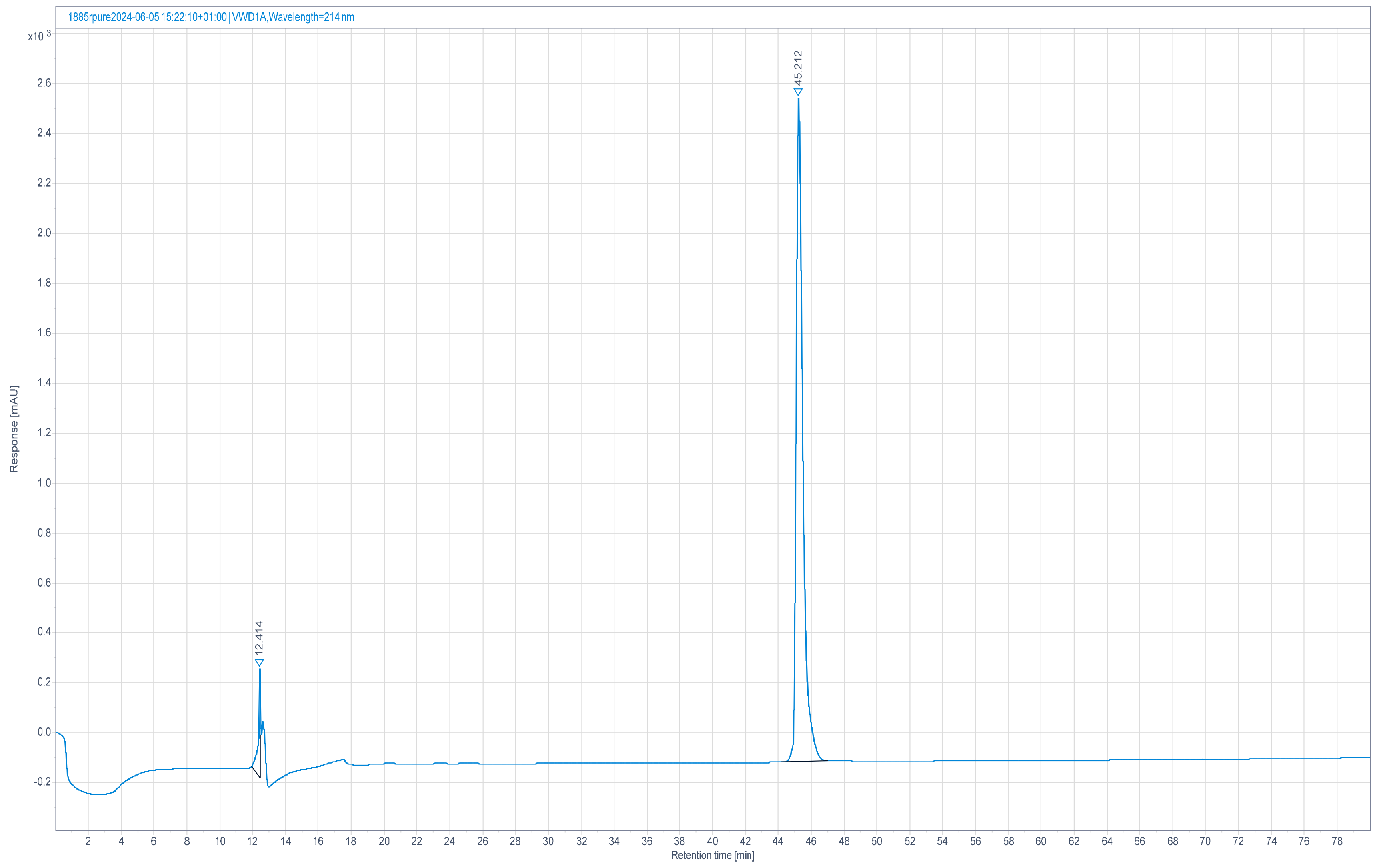
1885rpure2024-06-05 15:22:10+01:00 | VWD1A,Wavelength=214 nm
x10 3
Response [mAU]
Retention time [min]
12.414
45.212

1924a

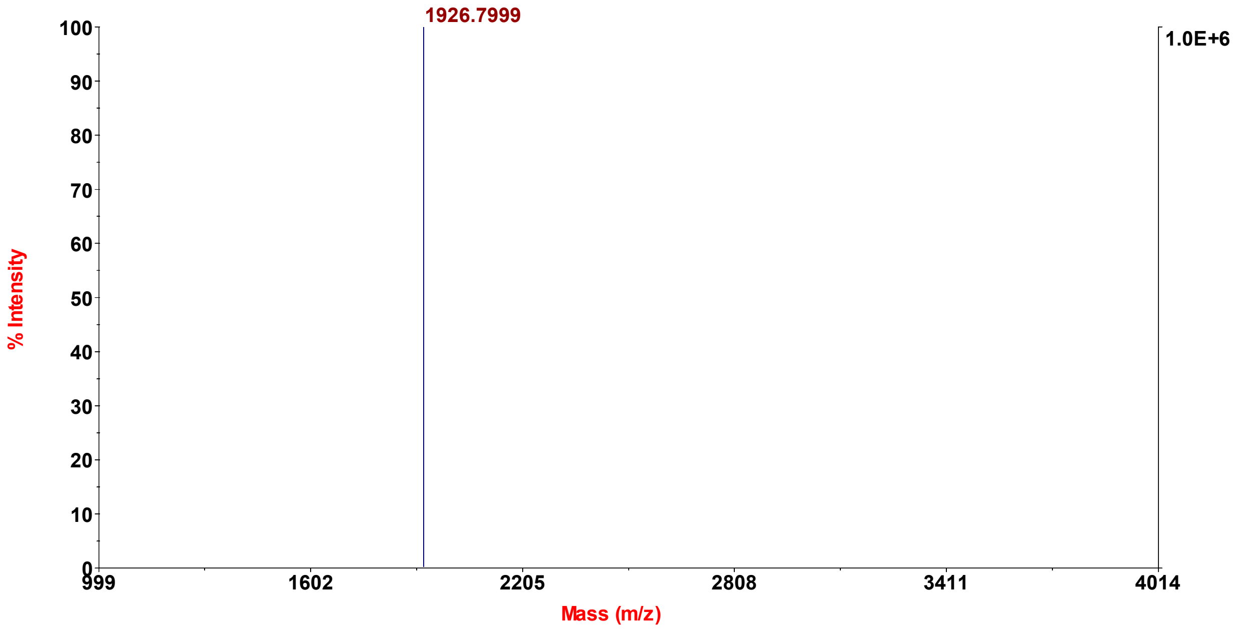
1926.7999
100
90
80
70
60
50
40
30
20
10
0
% Intensity
1.0E+6
999
1602
2205
2808
3411
4014
Mass (m/z)

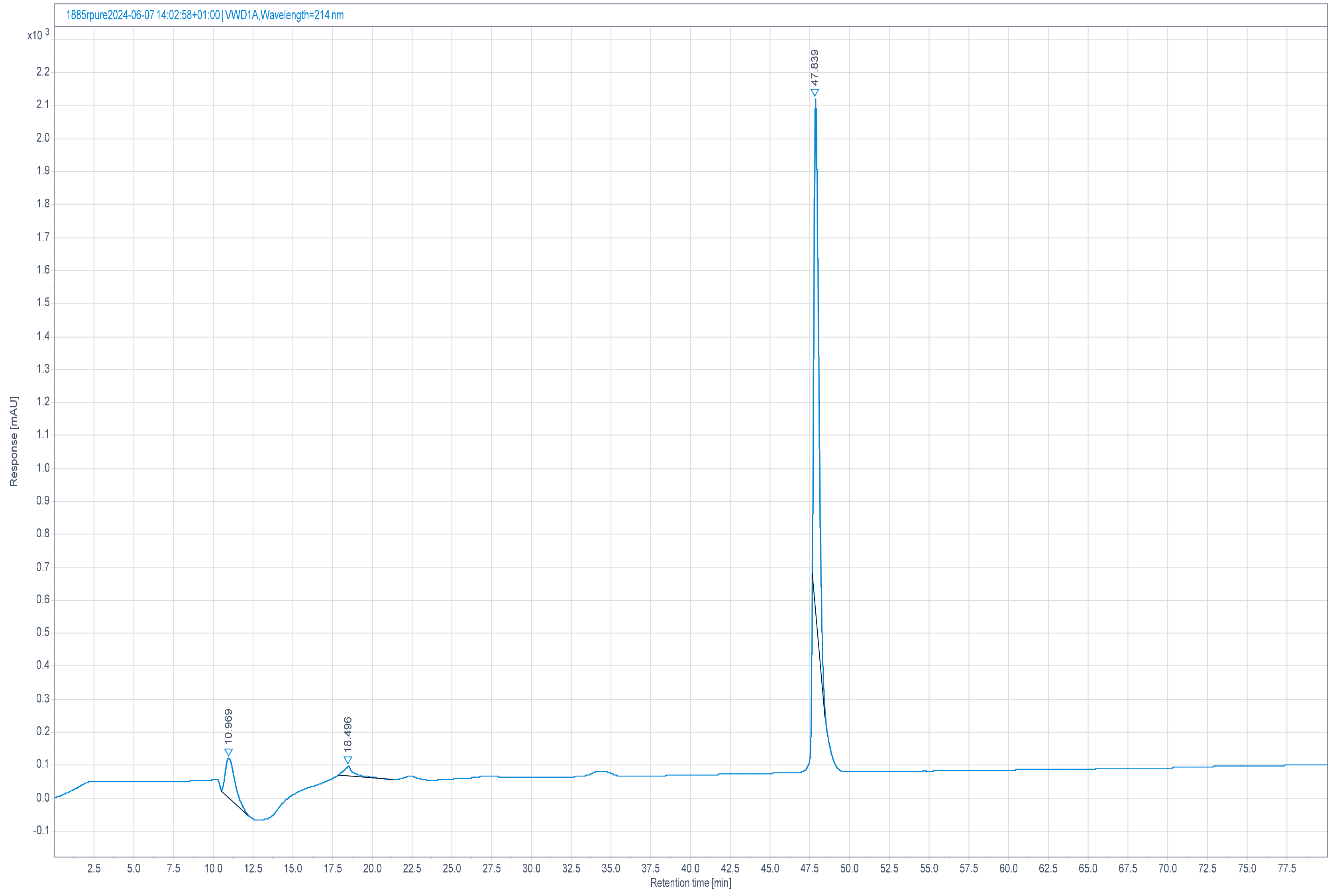
1885rpure2024-06-07 14:02:58+01:00 | VWD1A,Wavelength=214 nm
x10 3
Response [mAU]
Retention time [min]
10.969
18.496
47.839

2057c

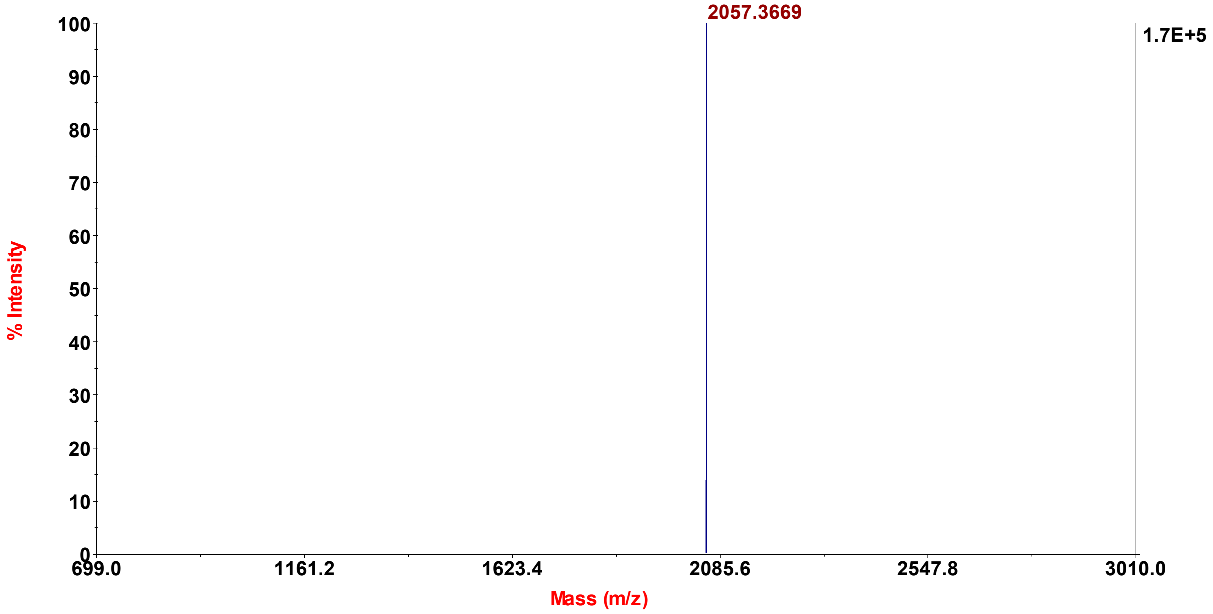

2057.3669
1.7E+5
100
90
80
70
60
50
40
30
20
10
0
% Intensity
699.0
1161.2
1623.4
2085.6
2547.8
3010.0
Mass (m/z)

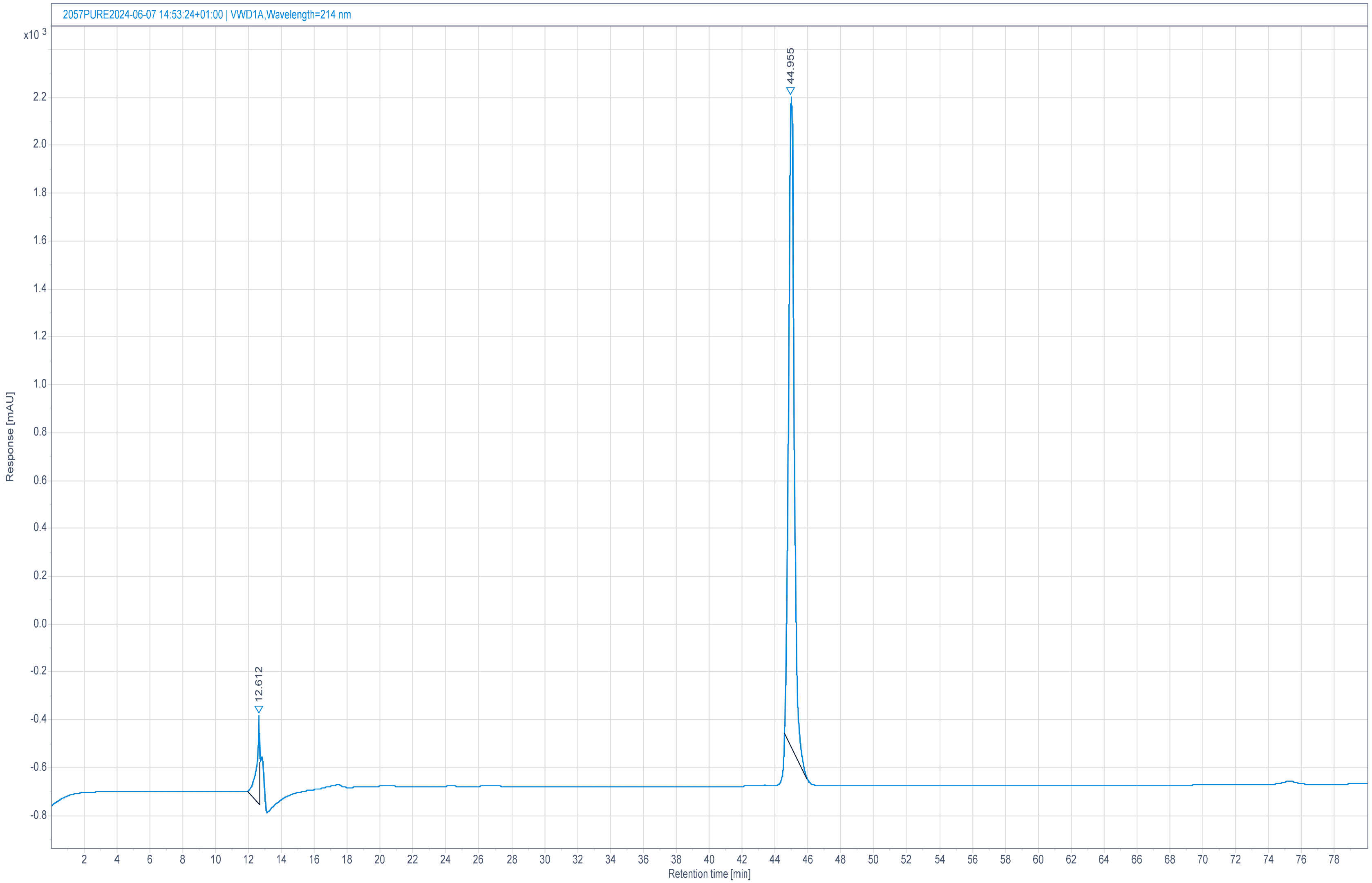
2057PURE2024-06-07 14:53:24+01:00 | VWD1A,Wavelength=214 nm
x10 3
Response [mAU]
Retention time [min]
12.612
44.955

2085-r

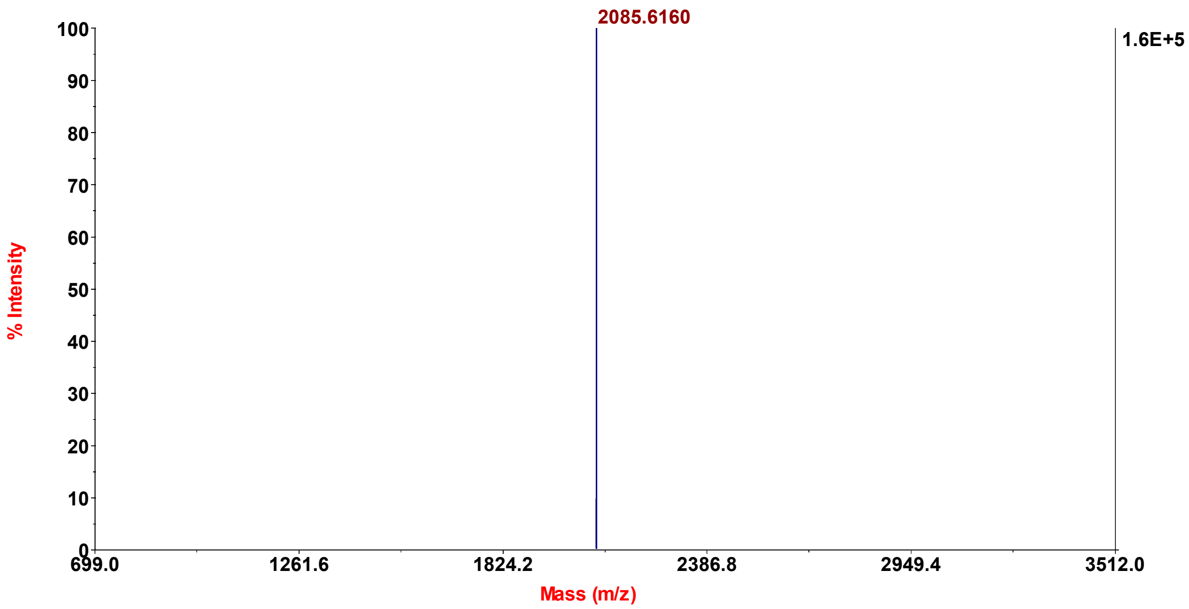

2085.6160
1.6E+5
100
90
80
70
60
50
40
30
20
10
0
% Intensity
699.0
1261.6
1824.2
2386.8
2949.4
3512.0
Mass (m/z)

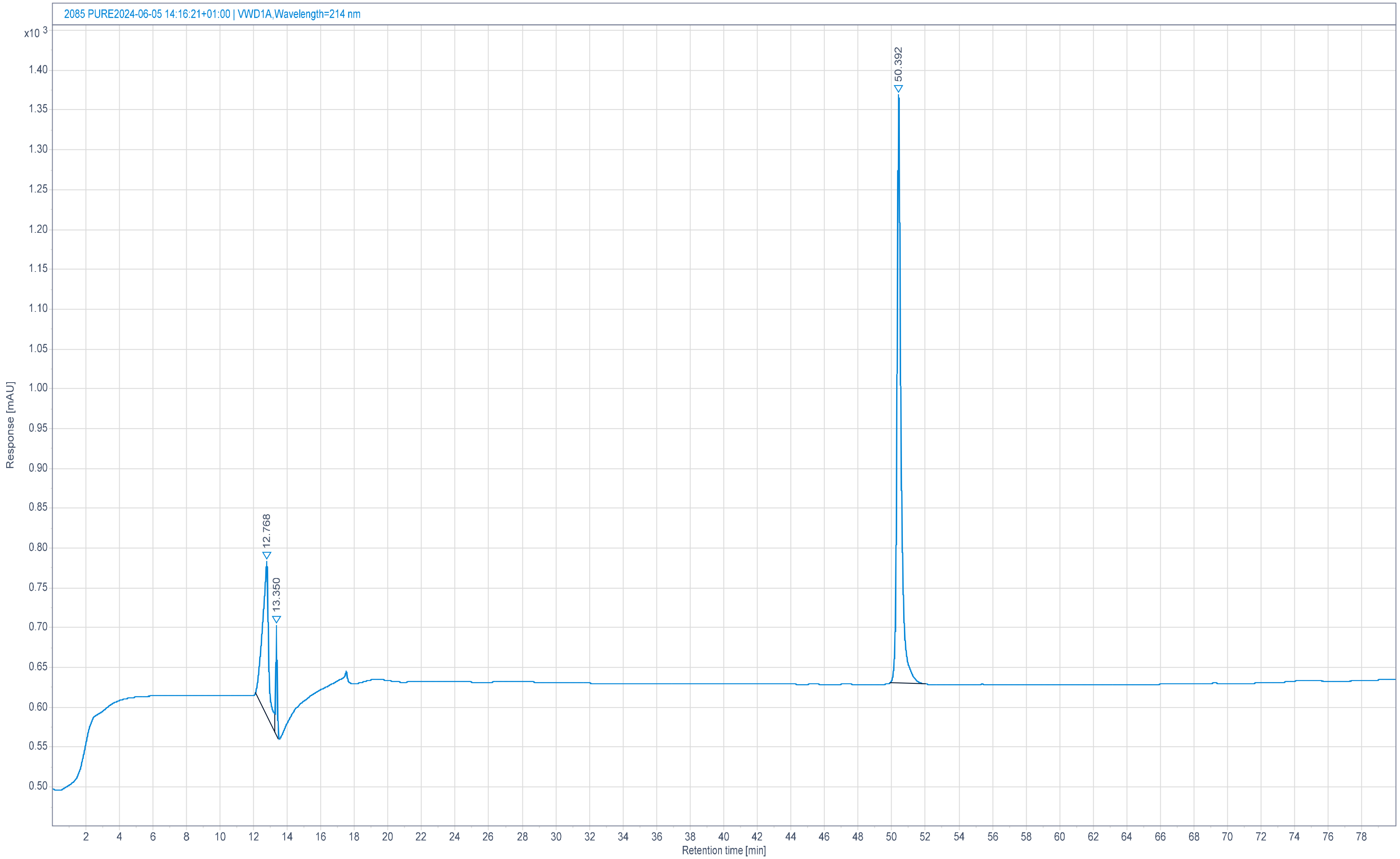
2085 PURE2024-06-05 14:16:21+01:00 | VWD1A,Wavelength=214 nm
x10 3
Response [mAU]
Retention time [min]
12.768
13.350
50.392

2170-2r

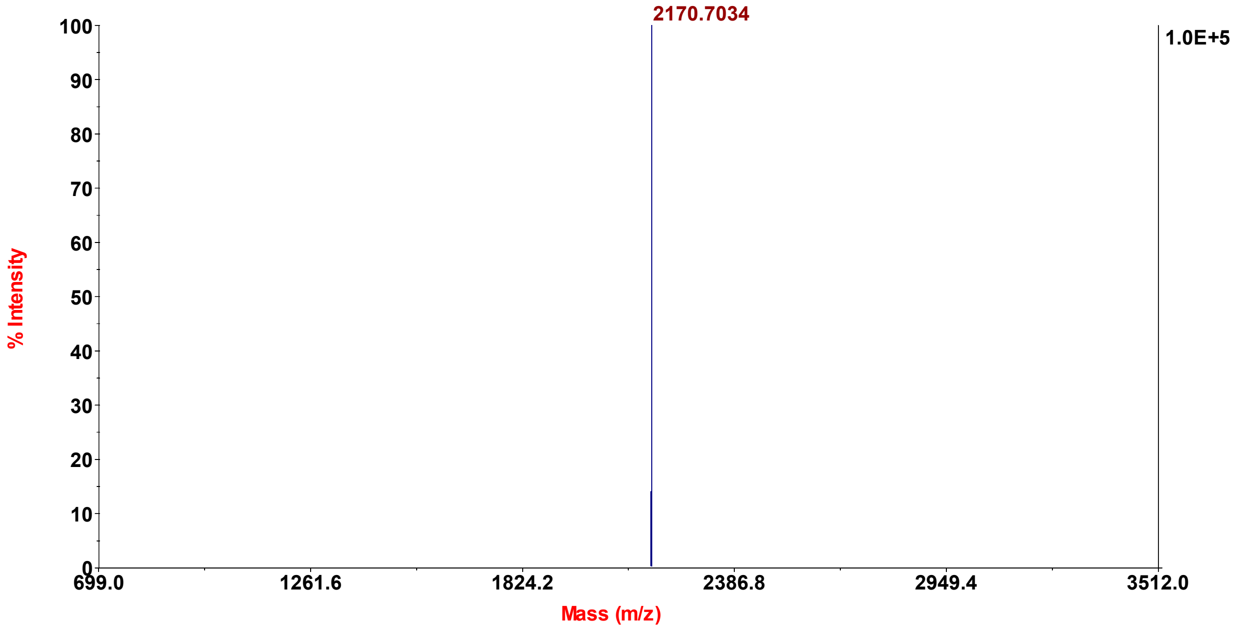

2170.7034
100
90
80
70
60
50
40
30
20
10
0
1.0E+5
% Intensity
699.0
1261.6
1824.2
2386.8
2949.4
3512.0
Mass (m/z)

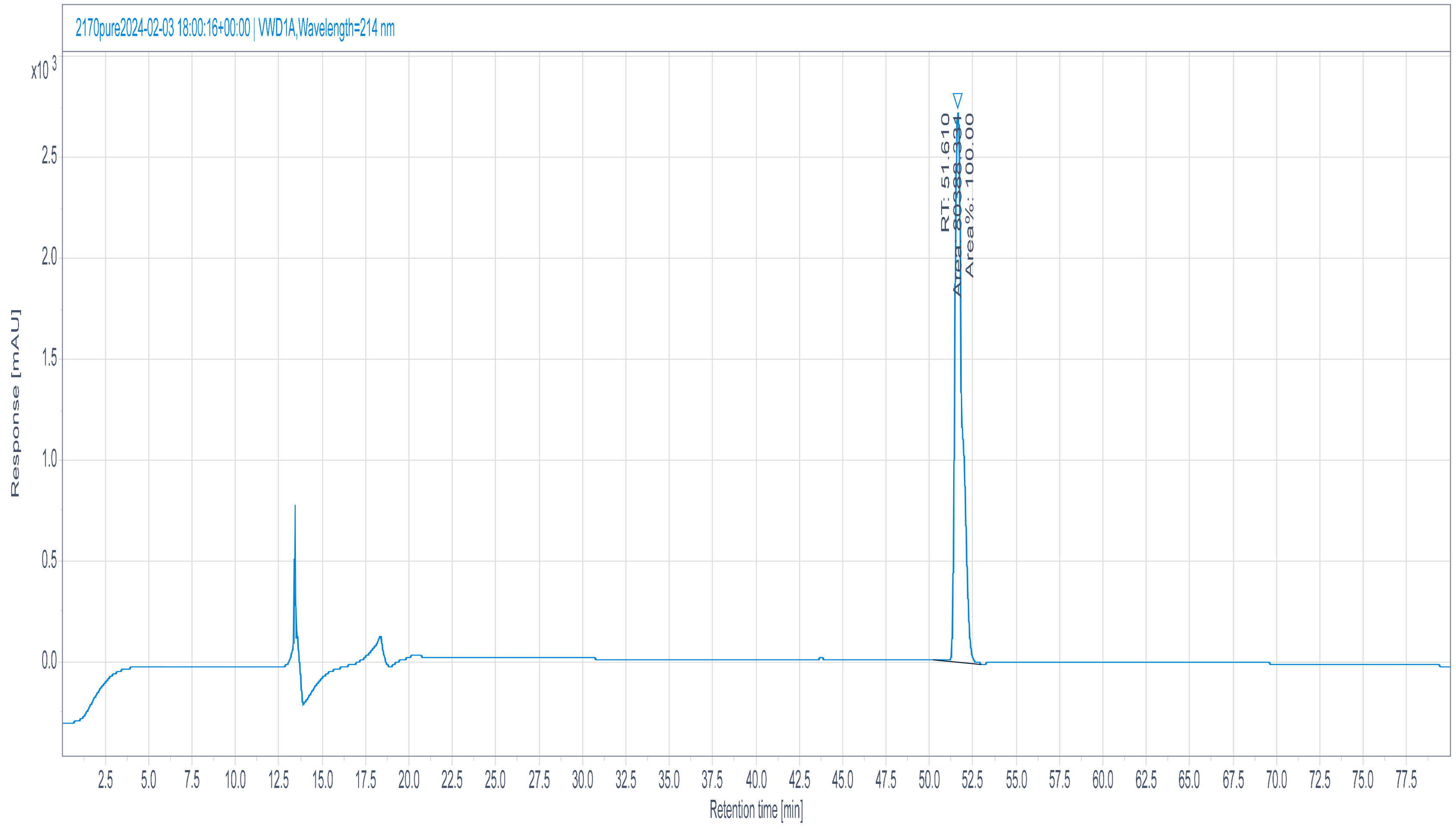
2170pure2024-02-03 18:00:16+00:00 | VWD1A,Wavelength=214 nm
x10 3
Response [mAU]
0.0
0.5
1.0
1.5
2.0
2.5
RT: 51.610
Area: 80388.334
Area%: 100.00
2.5
5.0
7.5
10.0
12.5
15.0
17.5
20.0
22.5
25.0
27.5
30.0
32.5
35.0
37.5
40.0
42.5
45.0
47.5
50.0
52.5
55.0
57.5
60.0
62.5
65.0
67.5
70.0
72.5
75.0
77.5
Retention time [min]

Figure S1. MALDI-TOF MS spectrum and purity of Nigrosin-6VL and its analogues.
